# High‐Tide Floods and Storm Surges During Atmospheric Rivers on the US West Coast

**DOI:** 10.1029/2021GL096820

**Published:** 2022-01-25

**Authors:** Christopher G. Piecuch, Sloan Coats, Sönke Dangendorf, Felix W. Landerer, J. T. Reager, Philip R. Thompson, Thomas Wahl

**Affiliations:** ^1^ Woods Hole Oceanographic Institution Falmouth MA USA; ^2^ University of Hawaii Honolulu HI USA; ^3^ Old Dominion University Norfolk VA USA; ^4^ Jet Propulsion Laboratory Pasadena CA USA; ^5^ University of Central Florida Orlando FL USA

**Keywords:** atmospheric rivers, high‐tide flooding, storm surge, coastal impacts, coastal hazards, sea level

## Abstract

Atmospheric rivers (ARs) cause inland hydrological impacts related to precipitation. However, little is known about coastal hazards associated with these events. We elucidate high‐tide floods (HTFs) and storm surges during ARs on the US West Coast during 1980–2016. HTFs and ARs cooccur more often than expected from chance. Between 10% and 63% of HTFs coincide with ARs on average, depending on location. However, interannual‐to‐decadal variations in HTFs are due more to tides and mean sea‐level changes than storminess variability. Only 2–15% of ARs coincide with HTFs, suggesting that ARs typically must cooccur with high tides or mean sea levels to cause HTFs. Storm surges during ARs reflect local wind, pressure, and precipitation forcing: meridional wind and barometric pressure are primary drivers, but precipitation makes secondary contributions. This study highlights the relevance of ARs to coastal impacts, clarifies the drivers of storm surge during ARs, and identifies future research directions.

## Key Points

- HTFs on the US West Coast cooccur with landfalling ARs more often than expected from random chance
- Between 10% and 63% of HTFs observed by tide gauges coincide with landfalling ARs, depending on location
- Meridional wind and barometric pressure make primary contributions to storm surge during ARs, but rainfall can also have a secondary effect

## 1 Introduction

Atmospheric rivers (ARs) are long, narrow filaments of strong horizontal water vapor transport in the lower troposphere, typically associated with cold fronts of extratropical cyclones (Cordeira et al., 2013; Ralph et al., 2004, 2017). ARs play an important role in the hydrological cycle, accomplishing most of the poleward moisture transport in the atmosphere at midlatitudes (Newman et al., 2012; Zhu & Newell, 1998). Landfalling ARs can be forced upwards by orography, leading to extreme precipitation and a range of hydrological impacts (Neiman et al., 2008). In California, for example, precipitation due to ARs has ended droughts and caused floods, landslides, and other debris flows (Dettinger, 2013; Du et al., 2018; Hendy et al., 2015; Oakley et al., 2017, 2018; Ralph et al., 2013; Wang et al., 2017; White et al., 2019).

While most studies of hazards related to ARs focus on hydrological impacts (Payne et al., 2020), the conditions typifying ARs—heavy rain, strong wind, low pressure—also drive storm surge at the coast (Gill, 1982; Pugh & Woodworth, 2014). Storm surge identifies an unusually high sea level above the predicted astronomical tide, generated by intense meteorological conditions usually experienced during storms. This suggests that ARs could be relevant to coastal impacts, such as high‐tide floods (HTFs). Also known as nuisance floods, sunny day floods, sea‐level rise floods, or recurrent tidal floods, HTFs refer to floods that occur when coastal still water levels exceed local minor flood thresholds, negatively affecting transportation, property, and public health and safety (Hino et al., 2019; Moftakhari et al., 2017, 2018; Sweet & Park, 2014; Sweet et al., 2021). The frequency of HTFs along the US West Coast has increased in recent decades in some places (San Diego, La Jolla, San Francisco, and Seattle), and more generally shows interannual variability that correlates with phases of the El Niño‐Southern Oscillation (ENSO; Sweet et al., 2021). However, few studies investigate the relationship between coastal sea level and ARs.

Khouakhi and Villarini (2016) quantify the correspondence between ARs and extreme sea‐level statistics on the US West Coast. They find that annual maxima of hourly still water levels at tide gauges between San Diego, California and Tofino, British Columbia occur within 12 hr of passing ARs 15–50% of the time. These authors also determine a relationship with modes of large‐scale climate variability. For example, exceedances over the 99.5th percentile of the hourly still water level distribution during ARs occur more frequently during El Niños and less frequently during La Niñas.

Shinoda et al. (2019) study the oceanic response to ARs during the CalWater 2015 field campaign. They observe daily averaged still water level anomalies of 30–50 cm at the Neah Bay, Washington and South Beach, Oregon tide gauges coinciding with landfalling ARs on January 16 and February 6, 2015. These authors determine that a high‐resolution ocean general circulation model reproduces the timing of observed storm surges, but only about half of their magnitude. Shinoda et al. (2019) posit that the storm‐surge response is mainly due to alongshore winds and coastal currents, and that model‐data discrepancies reflect small‐scale processes unresolved by the model.

These studies advance understanding of ARs and their impacts on sea level, but they also imply outstanding questions. First, the relationship between ARs and coastal impacts remains unclear. For instance, annual maxima and peaks‐over‐threshold statistics from Khouakhi and Villarini (2016) are not necessarily informative of HTFs. During years when no HTFs occur, annual maxima will not correspond to HTFs, whereas during years when multiple HTFs occur, some HTFs will not represent annual maxima. Likewise, the 99.5th percentile of a still water level distribution usually does not correspond to, and tends to be lower than, impact thresholds (Table S1 in Supporting Information S1; Sweet et al., 2018), meaning that many peaks‐over‐thresholds studied by Khouakhi and Villarini (2016) do not correspond to HTFs. Second, the factors driving storm surge during ARs remain to be established. For example, Shinoda et al. (2019) interpret storm surges during ARs in terms of the ocean's dynamic response to wind forcing. Their interpretation contrasts with Bromirski et al. (2017), who reason that the ocean's isostatic adjustment to barometric pressure is the primary mechanism of storm surge along the US West Coast. Khouakhi and Villarini (2016) recommend a future study to clarify the roles of wind and pressure forcing on storm surges during ARs.

Here we address these outstanding questions related to ARs, HTFs, and storm surges on the US West Coast. We consider tide‐gauge data, HTF thresholds, a catalog of ARs, and a gridded atmospheric reanalysis to establish the relationship between ARs and HTFs as well as the factors forcing storm surge during ARs. Results suggest that ARs contribute significantly to HTFs on the US West Coast, and clarify the relative effects of wind, pressure, and precipitation forcing on the associated storm surges.

## 2 Data

**Figure 1:**
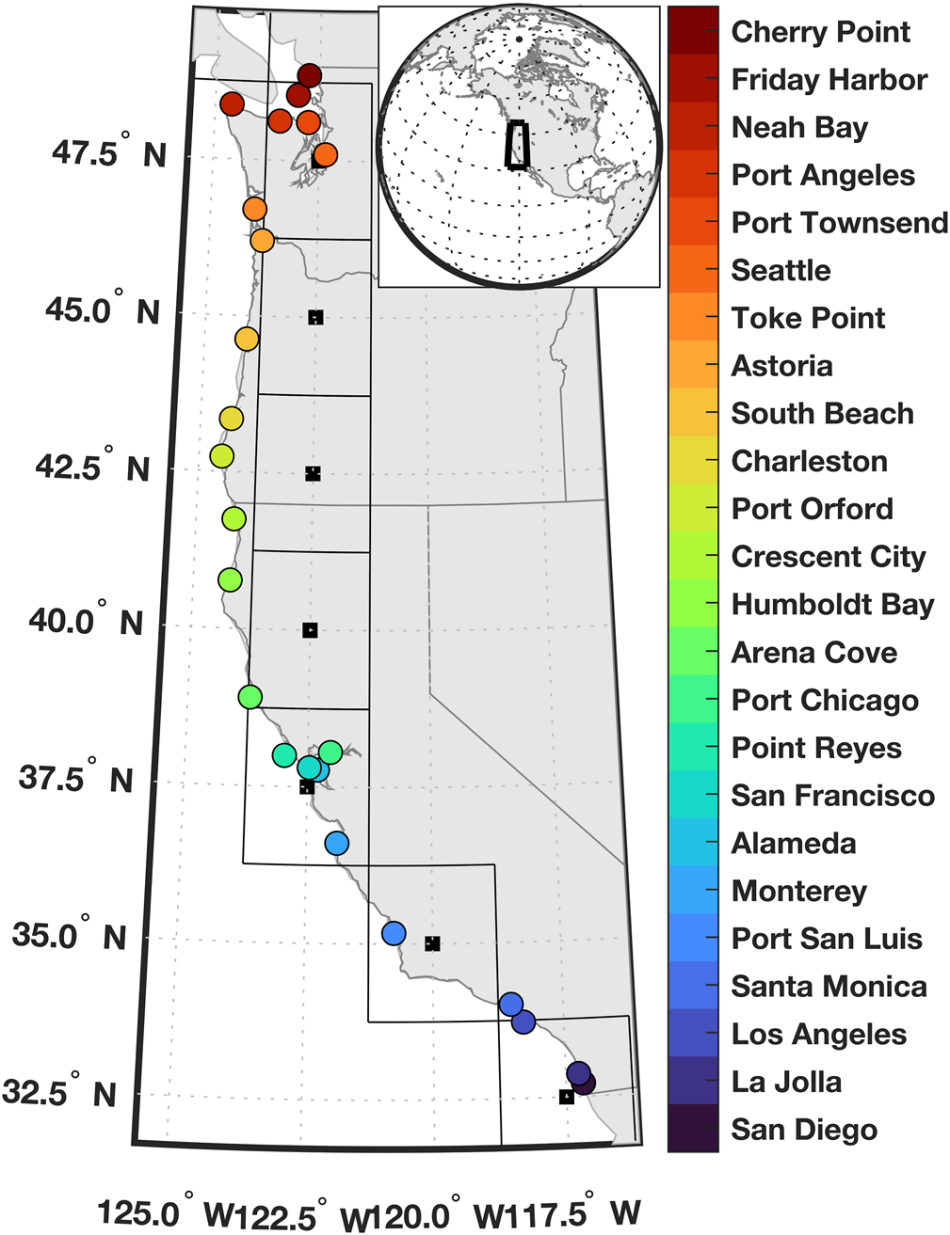
Study region. Colored circles identify locations of tide gauges. Thick black squares mark centers of grid cells in the catalog of Atmospheric rivers (ARs). Thin square outlines denote 2.5° × 2.5° catalog grid cell boundaries. Inset shows study region in global context.

We use hourly still water level observations, tidal predictions, and station datums for 24 tide gauges on the US West Coast from the National Oceanic and Atmospheric Administration (NOAA) Center for Operational Oceanographic Products and Services (CO‐OPS). These records are selected because they are relatively long, complete, and span much of the US West Coast (Figure 1 and Table S1 in Supporting Information S1). They also represent the union of US stations considered either in past studies of ARs and sea level on the US West Coast (Khouakhi & Villarini, 2016; Shinoda et al., 2019) or in government reports on HTFs (e.g., Sweet et al., 2021), allowing us to interpret our results in light of past findings.

We also use the Scripps Institution of Oceanography AR catalog of Gershunov et al. (2017), which is generated by applying an automated AR detection algorithm to 6‐hourly integrated water vapor transport (IVT) and integrated water vapor (IWV) from the National Centers for Environmental Prediction/National Center for Atmospheric Research (NCEP/NCAR) Reanalysis 1 (Kalnay et al., 1996). Landfalling ARs are identified by their spatial extent (≥1,500 km), temporal duration (≥18 hr), IVT (≥250 kg m^−1^ s^−1^), and IWV (≥15 mm). The landfalling location of an AR satisfying these criteria is defined as the reanalysis grid cell with the maximum IVT along the coast. The catalog includes the time, location, IWT, IVT, and zonal and meridional wind of ARs at their landfalling locations on a 2.5° × 2.5° grid along the US West Coast (22.5–57.5°N, 105–135°W; Figure 1) from January 1948 to March 2017. To complement information provided by the Gershunov et al. (2017) catalog, we also consider daily meridional and zonal wind stress, barometric pressure, and precipitation from the NCEP/NCAR Reanalysis 1. We also interrogated daily fields from the European Center for Medium‐Range Weather Forecasts (ECMWF) Reanalysis Interim (Dee et al., 2011) and obtained comparable results (Figures S1 and S2 in Supporting Information S1).

We consider the data between January 1, 1980 and December 31, 2016. The start date is chosen partly based on the tide‐gauge records, many of which begin in the late 1970s. By not considering data prior to 1980, we also avoid possible discontinuities in the reanalysis related to the advent of satellite data in the late 1970s. Data processing and methods specific to the analysis of either HTFs or storm surges are described in the next two sections before the respective results are introduced.

## 3 High‐Tide Floods

We establish relationships between ARs and HTFs on the US West Coast using a peaks‐over‐threshold approach (cf. Khouakhi & Villarini, 2016). For each tide gauge, we count the number of days when HTFs occur for at least 1 hr (HTF days). We identify HTFs when still water levels exceed the local minor flood thresholds defined by Sweet et al. (2018), which range between 56 and 64 cm above local mean higher high water (Table S1 in Supporting Information S1). We also count the number of days when an AR passes nearby a tide gauge (AR days). An AR is nearby a tide gauge when it has IVT ≥ 500 kg m^−1^ s^−1^ at its landfalling location in the grid cell whose centroid is closest to the gauge (Figure 1). Note that results are qualitatively insensitive to reasonable alternative definitions of “nearby” (Figures 3a and S7 in Supporting Information S1). We also count the number of days when both a HTF occurs and an AR passes nearby the gauge within ±24 hr of the HTF (HTF and AR days). Finally, we count the hypothetical number of days when HTFs would have occurred from mean sea‐level changes and tides alone by first calculating and removing storm surge from the hourly water level data, and then identifying days when the flood threshold is exceeded. (The calculation of storm surge is detailed in the next section.) For all quantities of interest, we run 1,000 bootstrap iterations to estimate uncertainty due to the finite record length of the data (Text S1 in Supporting Information S1). We quantify statistical significance of the observed numbers of HTF and AR days and other cooccurrences by comparing observed values to values determined synthetically through 1,000 simulations of uncorrelated stochastic processes (Text S2 in Supporting Information S1).

**Figure 2:**
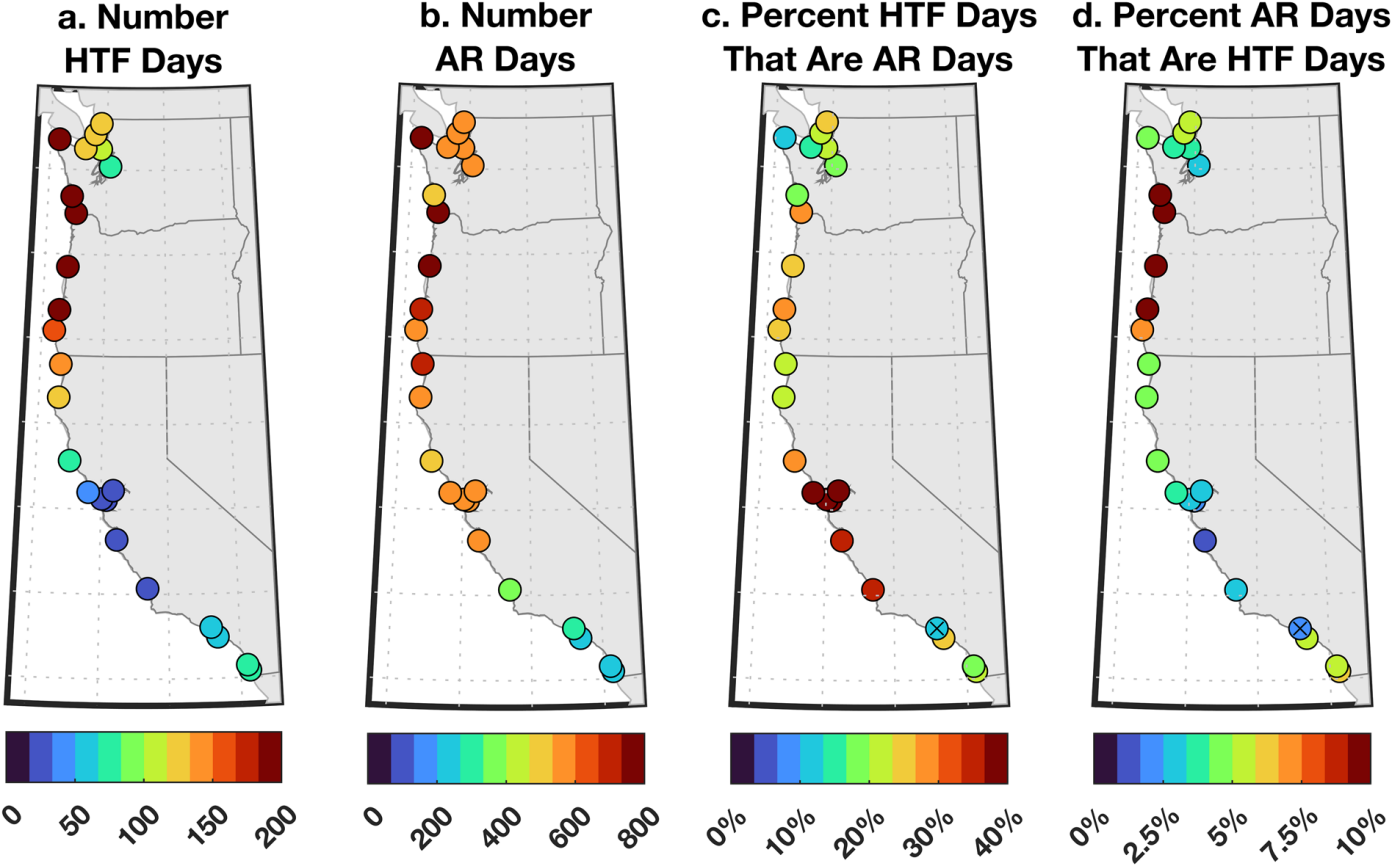
Number of (a) high‐tide flood (HTF) days and (b) atmospheric river (AR) days at tide gauges during 1980–2016. Percentage of (c) HTF days experiencing ARs, and (d) AR days experiencing HTFs. The “x” at Santa Monica, California in panels (c) and (d) indicates that the value is not significant given the null hypothesis of two uncorrelated stochastic Poisson processes (Text S2 in Supporting Information S1).

HTF days and AR days along the US West Coast show clear spatial structure (Figures 2a and 2b). More HTF days and AR days were experienced on the Northwest Coast than the Southwest Coast. For example, San Diego, California experienced 79 ± 17 HTF days and 259 ± 30 AR days during the study period, whereas Neah Bay, Washington witnessed 329 ± 37 HTF days and 760 ± 54 AR days over that same time. All ± ranges identify 95% confidence intervals based on bootstrapping. The Puget Sound in Washington is an exception to the rule: fewer HTF days and AR days occurred at higher‐latitude tide gauges in this estuary compared to lower‐latitude tide gauges on the open‐ocean coasts of Oregon and Washington, suggesting that these estuarine locations are more sheltered from processes driving HTFs and ARs. Central California also deviates from the trend, as fewer HTF days were observed at mid‐latitude locations in this region compared to lower‐latitude sites in Southern California. The basic patterns of HTF days and AR days found here are consistent with previous studies. For example, Sweet et al. (2021) report that more HTFs happen on the open coasts of Oregon and Washington than on the California coast or within the Puget Sound (their Appendix 1), while Neiman et al. (2008) find that more AR days occur on the Northwest Coast of North America than on the Southwest Coast. However, past studies do not interrogate possible connections between HTFs and ARs.

**Figure 3:**
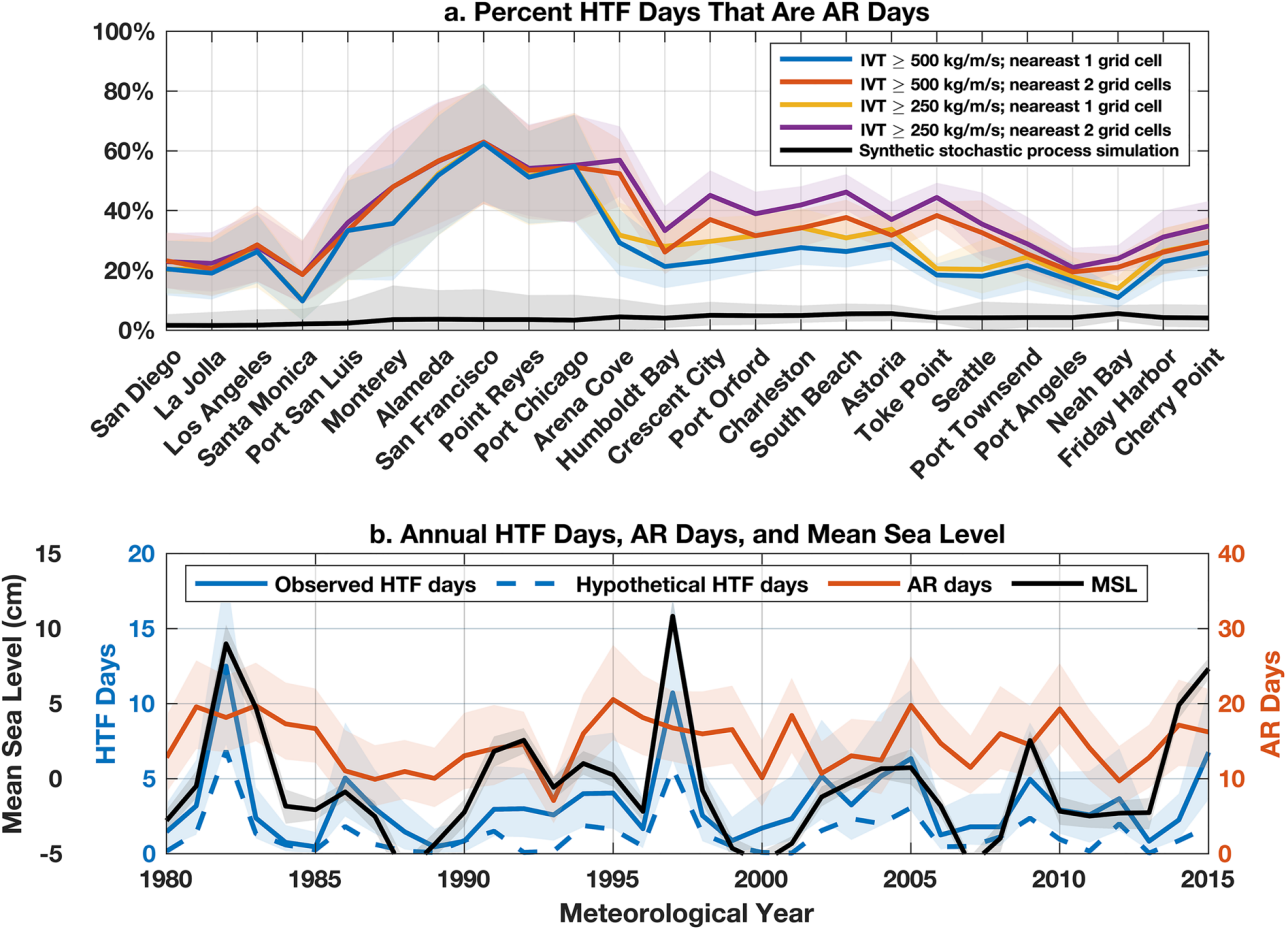
(a) Percentage of high‐tide flood (HTF) days with atmospheric rivers (ARs) during 1980–2016. Different colors identify different criteria applied to determine whether an AR is nearby during a HTF (i.e., whether the minimum IVT threshold is 250 or 500 kg m^−1^ s^−1^ and 1 or 2 nearby grid cells are considered). Black is the null hypothesis for two random stochastic Poisson processes (IVT ≥ 500 kg m^−1^ s^−1^ nearest 1 grid cell; Text S2 in Supporting Information S1). (b) Averages across all tide gauges along the US West Coast of yearly observed HTF days (blue), AR days (orange), and annual mean sea level (black). Thick lines and shaded values are, respectively, bootstrap estimates of average values and 95% confidence intervals. Blue dashed line is the best estimate of the number of HTF days per year expected hypothetically from tides and mean sea‐level changes (see text for details). Note that the horizontal axis has units of meteorological years (May–April).

To clarify relationships between ARs and HTFs, we compute percentages of HTF days that are AR days and AR days that are HTF days (Figures 2c, 2d and 3a). The percentage of HTF days that are AR days quantifies whether ARs are a necessary condition for HTFs (values ∼100% indicate that HTFs only occur during ARs), while the percentage of AR days that are HTF days measures whether ARs are a sufficient condition for HTFs (values ∼100% indicate that ARs always lead to HTFs). On average along the coast, 28% ± 2.3% of HTF days are AR days, but values are elevated between Monterey and Arena Cove (48% ± 6.9%) in Central California, with the highest percentage (63% ± 19%) observed at San Francisco (Figures 2c and 3a). Similar findings were obtained using alternative criteria for evaluating if an AR is near a tide gauge (Figures 3a and S7 in Supporting Information S1), meaning that these results are robust to these subjective analysis choices. In comparison, since more AR days occurred than HTF days (Figures 2a and 2b), the percentage of AR days that are HTF days is lower on average (5.2% ± 0.4%), peaking more to the north, with 10% ± 1.1% of AR days being HTF days between Port Orford, Oregon and Toke Point, Washington (Figure 2d), suggesting that ARs alone are seldom sufficient to cause HTFs. Nevertheless, at nearly all sites, values in Figures 2c, 2d and 3a are statistically significant (*p* < 0.05), meaning that HTFs and ARs cooccur more often than expected from random chance, and suggesting that ARs are important contributors to HTFs.

HTF and AR frequencies also vary across time (Figure 3b). The annual number of HTFs averaged along the US West Coast varies from 0.7 ± 0.7 to 13 ± 5.9 days per year, while the average number of ARs ranges between 7.2 ± 3.1 and 21 ± 6.3 days per year (Figure 3b). HTF days were highest in 1982 (13 ± 5.9 days) and 1997 (12 ± 5.4 days) during strong positive ENSO events. This observation is consistent with past studies identifying a relationship between ENSO and HTF frequency on the US West Coast (Sweet & Park, 2014; Sweet et al., 2021). The Pearson correlation coefficient between interannual variations in HTF and AR days on the US West Coast (0.2 ± 0.2) is not statistically significant (*p* > 0.05). In contrast, the number of HTF days per year is significantly correlated with annual mean sea‐level anomaly averaged along the coast (0.7 ± 0.1, *p* < 0.01; Figure 3b). An even higher correlation (0.9 ± 0.1, *p* < 0.01) is found between observed HTF days and hypothetical HTF days expected from tides and mean sea‐level changes, such that the latter explains 66 ± 14% of the variance in the former, suggesting that changes in these extreme sea‐level events are governed more by tides and mean sea‐level changes than changes in storminess (cf. Marcos et al., 2015; Menéndez & Woodworth, 2010; Ray & Merrifield, 2019; Thompson et al., 2021).

## 4 Storm Surges

We quantify storm surges and their causes during ARs on the US West Coast using a composite analysis (cf. Shinoda et al., 2019). We identify all ARs passing by tide gauges during the study period. For each AR as it passes by a gauge, we isolate the day when maximum IVT takes place and interpret it as when the gauge experiences the strongest effect of the AR. We then take the associated daily storm surge from the tide gauge, which we calculate from daily mean still water level by removing the tidal prediction, seasonal cycle, and linear trend, and then applying a high‐pass filter based on a 20‐day moving‐median operator. We use the 20‐day time scale to separate higher‐frequency storm surges from lower‐frequency changes in mean sea level, similar to past studies (Dangendorf et al., 2016; Serafin et al., 2017). The moving median represents a robust tool for detecting extremes in the presence of trends and other noise (Mudelsee, 2020).

**Figure 4:**
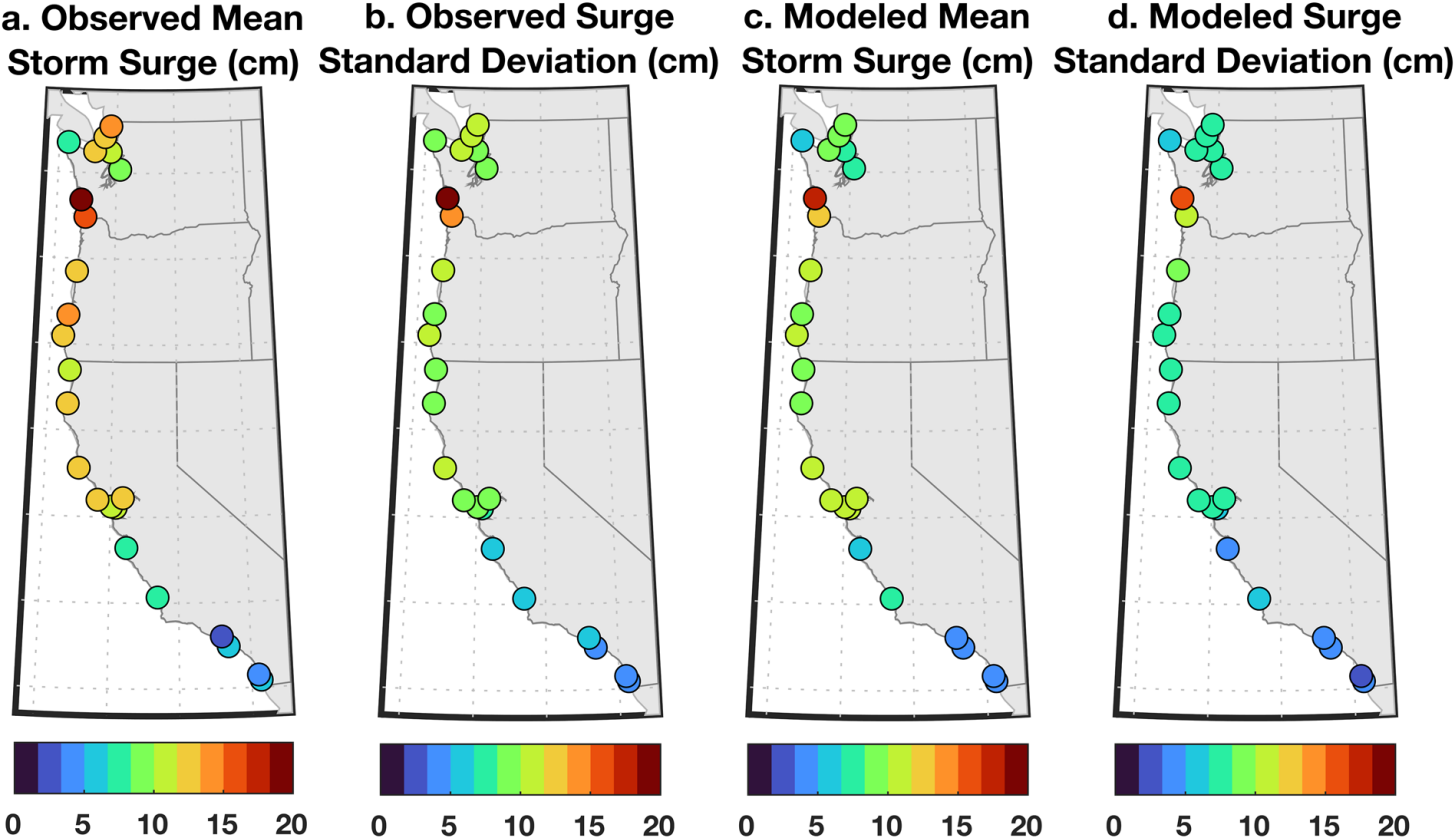
Composite (a) averages and (b) standard deviations of storm surge during atmospheric rivers (ARs) observed by tide gauges over 1980–2016. (c, d) As in (a, b) but based on the ridge‐regression model including local wind, pressure, and precipitation forcing.

**Figure 5:**
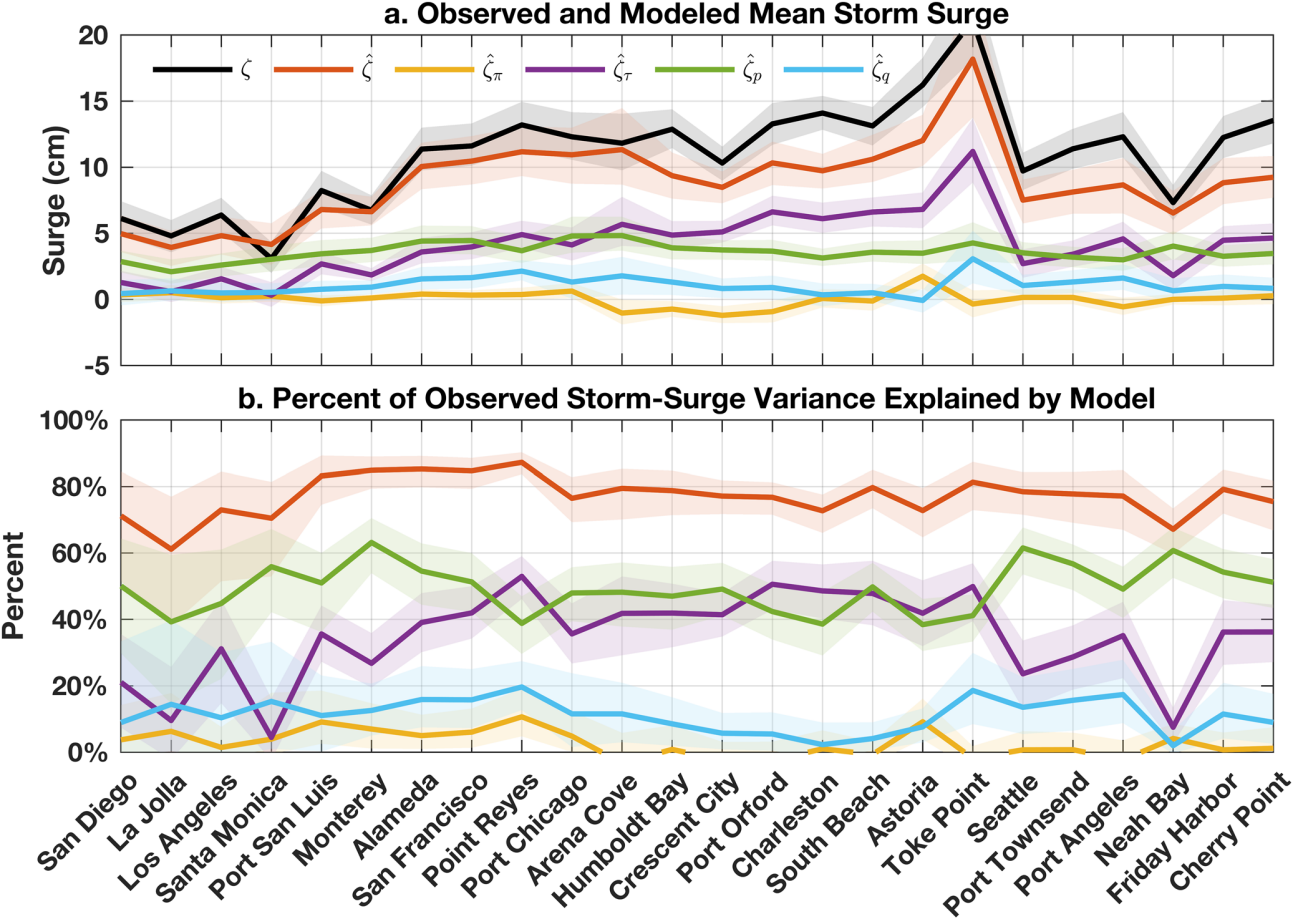
(a) Average *ζ* value across all atmospheric rivers (ARs) observed by tide gauges during 1980–2016 (black) alongside corresponding total $\hat{\zeta }$ (orange), zonal‐wind‐driven ${\hat{\zeta }}_{\pi }$ (yellow), meridional‐wind‐driven ${\hat{\zeta }}_{\tau }$ (purple), pressure‐driven ${\hat{\zeta }}_{p}$ (green), precipitation‐driven ${\hat{\zeta }}_{q}$ (blue) modeled values. (b) Observed *ζ* variance explained by the various model estimates at each tide gauge during 1980–2016. Thick lines and shaded values are, respectively, bootstrap estimates of the mean and 95% confidence interval.

Storm surges during ARs show clear spatial structure (Figures 4a, 4b and 5a). Surges are larger on average at higher latitudes (Figures 4a and 5a). Mean storm surge during an AR grows from 3.1 ± 1.2 cm at Santa Monica, California to 21 ± 3.2 cm at Toke Point, Washington. Deviations from this trend are apparent at locations in the Puget Sound, where mean surge values are lower than expected from latitude alone, which could reflect important estuarine processes distinct from the mechanisms that mediate storm surge along the open‐ocean coastline. Storm surge is also more variable at higher latitudes (Figure 4b). For example, the standard deviation of storm surge during ARs is 4.3 ± 0.8 cm at La Jolla, California, 12 ± 1.6 cm at South Beach, Oregon, and 20 ± 5.3 cm at Toke Point, Washington. [Note that, while we use mean and standard deviation as summary statistics, storm surge distributions are not Gaussian (Figure S3 in Supporting Information S1).] Such surges are rarely large enough, when superimposed on mean higher high water, to overtop flood thresholds (cf. Table S1 and Figure S3 in Supporting Information S1). This corroborates the suggestion made in the previous section that ARs alone are seldom sufficient to cause HTFs.

These basic patterns are qualitatively consistent with previous numerical studies of sea level and ARs as well as past observational studies of storm surge in the region. Considering tide‐gauge data during 1935–2014, Bromirski et al. (2017) show that the 99th percentile of hourly nontidal winter residuals increases steadily from 10 to 15 cm in Southern California to 45–55 cm in Oregon and Washington (their Figure 2c). Serafin et al. (2017) reveal that the average and spread of observed annual maxima in hourly nontidal residuals from 11 tide gauges between La Jolla, California and Neah Bay, Washington, increase from south to north along the coast (their Figure 1e). Using a high‐resolution ocean general circulation model, Shinoda et al. (2019) report that coastal sea level rises during the days leading up to an AR by between ≲1 cm off Southern California to ≳4 cm off Oregon and Washington (their Figure 8h). However, these studies do not establish what processes drive storm surge during landfalling ARs.

To attribute observed surges (Figures 4a and 4b), we use contemporaneous daily zonal and meridional wind stress, barometric pressure, and precipitation from NCEP/NCAR Reanalysis 1 at the grid cells closest to the tide gauges. We remove seasonal cycles and linear trends from the reanalysis and apply a 20‐day high‐pass filter. To quantify how much storm surge can be understood in terms of local wind, pressure, and precipitation anomalies, we consider a simple model that represents surge as a linear superposition of the atmospheric forcing

(1) $$\zeta =\overset{\hat{\zeta }}{\overset{︷}{\underset{{\hat{\zeta }}_{\pi }}{\underset{︸}{a_{\pi }\pi +b_{\pi }H\left(\pi \right)}}+\underset{{\hat{\zeta }}_{\tau }}{\underset{︸}{a_{\tau }\tau +b_{\tau }H\left(\tau \right)}}+\underset{{\hat{\zeta }}_{p}}{\underset{︸}{a_{p}p+b_{p}H\left(p\right)}}+\underset{{\hat{\zeta }}_{q}}{\underset{︸}{a_{q}q+b_{q}H\left(q\right)}}}}+\epsilon ,$$

where *ζ* is storm surge [in m], *π* and *τ* are zonal and meridional wind stress [N m^−2^], respectively, *p* is barometric pressure [Pa], *q* is precipitation [m s^−1^], *ϵ* is a residual [m], H is Hilbert transform, and *a*'s and *b*'s are real constants. The form of Eq. (1) reflects physical considerations. On these time scales, we anticipate a transient adjustment, such that the ocean's response may be out of phase with the atmospheric forcing (Gill, 1982). The Hilbert transform, or quadrature function, rotates each Fourier component of a time series by ±90° (Thomson & Emery, 2014). Therefore, by including forcing terms and their Hilbert transforms in Equation 1, we represent arbitrary phase relationships between forcing and response. In contrast, a regression model that included the forcing terms but omitted their Hilbert transforms would only permit in‐phase or antiphase relationships. For clarity, let ${\hat{\zeta }}_{\pi }$, ${\hat{\zeta }}_{\tau }$, ${\hat{\zeta }}_{p}$, and ${\hat{\zeta }}_{q}$ identify the modeled *ζ* responses to *π*, *τ*, *p*, and *q* forcing, respectively, and $\hat{\zeta }$ the total modeled *ζ* response. We use ridge regression to determine the *a*'s and *b*'s at each tide gauge (Text S3 in Supporting Information S1), which is preferable to ordinary least squares given possible collinearity between predictors. Results are based on a ridge‐parameter value of *λ* = 0.3, but similar findings follow from a range of *λ* values (Figure S4 in Supporting Information S1).

Modeled $\hat{\zeta }$ shows skill in explaining *ζ* observed at tide gauges (Figures 4 and 5). The model reproduces the observed structure that surges grow larger and more variable with latitude along the coast (Figure 4). Mean storm surges from the observations *ζ* and the model $\hat{\zeta }$ overlap within estimated uncertainties everywhere on the California coast (Figure 5a). Along Oregon and Washington, the model can underestimate observed mean storm surge (by as much as 32% on average at Cherry Point, Washington), possibly due to shrinkage related to the ridge regression, reanalysis errors (e.g., due to coarse grid cells that overlap land and sea), or processes absent from the model (Figure 5a). The model also accounts for most of the observed storm‐surge variation at all gauges (Figure 5b), explaining between 57 ± 20% (La Jolla, California) and 87 ± 3.4% (Point Reyes, California) of the variance in the data, where the percent variance V in a variable *x* explained by another variable *y* is defined as

(2) $$V=100\%\times \left[1-\frac{var\left(x-y\right)}{var\left(x\right)}\right],$$

and var is the variance operator. As defined in Equation (2), V is similar to the familiar Nash‐Sutcliffe model efficiency coefficient from hydrology (Gupta et al., 2009; Li, 2016; Nash & Sutcliffe, 1970).

The model is also informative of the relative influences of *π*, *τ*, *p*, and *q* forcing on *ζ* (Figure 5). Primary contributions to *ζ* are made by *p* and *τ* (Figure 5). On average, ${\hat{\zeta }}_{p}$ contributions to mean *ζ* values are nearly spatially uniform along the coast, ranging between 2 and 5 cm (Figure 5a). In contrast, average ${\hat{\zeta }}_{\tau }$ values become larger with latitude, growing from 0.3 ± 0.9 cm at Santa Monica, California to 11 ± 2.3 cm at Toke Point, Washington. In Southern California and within Puget Sound, ${\hat{\zeta }}_{p}$ is the more important contributor to *ζ* variance, but elsewhere ${\hat{\zeta }}_{\tau }$ and ${\hat{\zeta }}_{p}$ contribute comparably (Figure 5b). Forcing by *q* can also make secondary contributions (Figure 5). Mean ${\hat{\zeta }}_{q}$ values are distinguishable from zero at most sites, reaching as high as 2.3 ± 0.8 cm in Point Reyes, California and 3.2 ± 2.0 cm in Toke Point, Washington (Figure 5a). In and around San Francisco Bay, and along portions of the Washington coast, ${\hat{\zeta }}_{q}$ explains 10–20% of the *ζ* variance on average (Figure 5b). In contrast, *π* forcing is largely insignificant (Figure 5). In most places, estimates of *ζ* variance explained by ${\hat{\zeta }}_{\pi }$ overlap with zero (Figure 5b), and mean ${\hat{\zeta }}_{\pi }$ values are indistinguishable from zero or small and negative (Figure 5a). Two reasons may together explain our finding that *π* is not an important contributor. First, *τ* anomalies, which are mostly alongshore on the US West Coast, are typically stronger than *π* anomalies, which are mainly onshore, during ARs at gauges (Figure S6 in Supporting Information S1). Second, *ζ* can be more sensitive to an alongshore wind stress anomaly than to an onshore wind stress anomaly of equal magnitude (Text S4 and Figures S4, S5 in Supporting Information S1). More generally, spatial structures apparent in storm‐surge contributors (Figure 5) may have partly to do with variations in the orientation or strength of ARs along the coast.

## 5 Summary and Discussion

Atmospheric rivers (ARs) typically bring heavy rain, strong wind, and low pressure to the coastal zone. We established relationships between ARs and high‐tide floods (HTFs), and identified forcing mechanisms responsible for storm surge during ARs on the US West Coast during 1980–2016. ARs and HTFs cooccur more often than expected from random chance, and 10–63% of HTFs coincide with ARs, depending on location (Figures 2 and 3). Interannual variations in HTF days and AR days per year are not significantly correlated (Figure 3), meaning that more ARs do not necessarily result in more HTFs. Instead, there is a significant correlation between observed HTF days per year and the HTF days expected from tides and mean sea‐level changes alone (Figure 3). A linear model including local wind, pressure, and precipitation forcing accounts for ≥68% of the average magnitude and 57–87% of the variance in magnitude of storm surges during ARs (Figures 4 and 5). Meridional wind and barometric pressure make primary contributions to storm surge, but precipitation has a secondary effect in some places (Figure 5).

HTFs arise from a subtle interplay of distinct processes acting on different timescales. While they tend to occur and be most acute at high tide, HTFs are generally not caused by tides alone (cf. Hague & Taylor, 2021). Fewer HTFs would occur on average from tides and mean sea‐level changes in the absence of surges due to ARs and other events (Figure 3). However, surges associated with ARs are rarely large enough, when added to mean higher high water, to cause HTFs on their own (Figure 3). It is only when superimposed on a favorable tide or mean sea‐level anomaly that storm surges related to ARs are generally capable of exceeding HTF thresholds. For a full understanding of observed HTFs, the effects of surges, tides, and mean sea level must all be considered.

This paper advances knowledge of hazards related to ARs and the oceanic response to atmospheric forcing on the US West Coast. Past studies emphasize hydrological impacts of ARs related to extreme precipitation (Payne et al., 2020), but we show that ARs also drive coastal impacts related to sea level. By quantifying relationships between HTFs and ARs, and identifying the factors driving storm surge during these events, we resolve outstanding questions in the literature (Bromirski et al., 2017; Khouakhi & Villarini, 2016; Shinoda et al., 2019). This paper sheds light on HTFs, occurrences of which are increasing on much of the US Coast (Sweet et al., 2021), and will accelerate into the future (Thompson et al., 2021). Our work is consistent with the notion that observed changes in sea‐level extremes are attributable more to changes in mean sea level and the tides than to changes in storminess (Marcos et al., 2015; Menéndez & Woodworth, 2010; Ray & Merrifield, 2019; Thompson et al., 2021). Our results also underscore the importance of understanding locally forced high‐frequency sea‐level variability on the US West Coast (Battisti & Hickey, 1984; Bromirski et al., 2017; Chapman, 1987; Gill & Clarke, 1974; Ryan & Noble, 2006; Verdy et al., 2014).

We conclude with some limitations of our study and future research directions.

1. Space constraints precluded a complete study of the spatiotemporal statistics of HTFs and ARs on the US West Coast. Future studies should consider more granular details, such as temporal variation in HTF and AR cooccurrences at individual tide gauges across various timescales, including the seasonal cycle and decadal trends, to identify whether sea‐level rise influences the covariance between HTFs and ARs, and if HTFs due to ARs occur mainly in particular months of the year (Thompson et al., 2021).
2. We focused on the US West Coast, but ARs make landfall in other midlatitude and high‐latitude regions (Payne et al., 2020). Links should be established between ARs and sea‐level extremes on a more global basis (cf. Carvajal et al., 2021; Ridder et al., 2018).
3. We used the AR catalog of Gershunov et al. (2017), but other AR data sets are available, which differ in terms of their detection algorithms (Rutz et al., 2019; Shields et al., 2018). These differences can affect the frequency, intensity, and duration ARs identified by the data sets (Ralph et al., 2019). For example, considering a catalog using more selective criteria that recognized fewer, more intense AR events, we may find higher average storm surge (cf. Figures 4 and 5) but smaller percentage of HTF days that are AR days (cf. Figures 2 and 3). A thorough error analysis is beyond our scope, but we considered 22 other AR data sets participating in the “Tier 1” Atmospheric River Tracking Method Intercomparison Project (ARTMIP; Text S5 in Supporting Information S1). Findings based on the Gershunov et al. (2017) catalog are consistent with and representative of results obtained from the other ARTMIP data sets more generally (Figure S7 in Supporting Information S1). This suggests that, had we used another AR catalog for our main analysis, we would expect similar results on average. However, uncertainties on the estimates from the various ARTMIP data sets can be large (Figure S7 in Supporting Information S1). A future study should identify the origins of these uncertainties and what AR catalogs are most informative for studies of coastal impacts.
4. We focused on storm surge and HTFs, but ARs could affect other quantities of interest to coastal impacts, such as waves and erosion (Serafin et al., 2017; Theuerkauf et al., 2014). A more comprehensive assessment of coastal hazards due to landfalling ARs, including their role in compound events (AghaKouchak et al., 2020), should be made.
5. We used flood thresholds from the common impact threshold framework of Sweet et al. (2018), which is a consistent national coastal flood metric, applicable everywhere tidal datums are established. However, flood thresholds based on this framework may be lower or higher than levels that correspond to local impacts (Kriebel & Geiman, 2013). The sensitivity of our results to other definitions of flood threshold should be quantified.
6. Our investigation of storm surge was statistical in nature. Regression coefficients found empirically from the data are consistent with basic expectations for a coastal‐trapped barotropic sea‐level response to local wind, pressure, and precipitation forcing over a frictional shelf (Text S4 and Figure S4 in Supporting Information S1), suggesting that we identify causal relationships between storm surge and atmospheric forcing. Even so, a more physics‐based assessment would be informative, allowing the relative roles of the various (correlated) forcing mechanisms to be more unambiguously identified. Because precipitation is not often identified as a driver of storm surge (Gill, 1982; Pugh & Woodworth, 2014), it would be particularly informative to test our hypothesis that precipitation can contribute to storm surge during ARs.
7. We used observations of the past four decades, but the nature of ARs could change under future warming. While their dynamical response to climate change remains uncertain (Shepherd et al., 2014; Vallis et al., 2015), ARs are expected to become more frequent (Espinoza et al., 2018), contain more moisture (Dettinger, 2011), and shift poleward (Yin, 2005) as the climate changes. It remains to evaluate how future changes in ARs would aggravate coastal impacts already expected from future sea‐level rise (Jevrejeva et al., 2019; Kopp et al., 2017).

## Supporting information

Supporting Information S1

## Data Availability

Tide‐gauge data, tidal predictions, and station datum information are from the NOAA Tides and Currents Service (https://tidesandcurrents.noaa.gov/). Reanalysis fields are from the NOAA Physical Sciences Laboratory (https://psl.noaa.gov/data/gridded/data.ncep.reanalysis.html) and ECMWF (https://www.ecmwf.int/en/forecasts/datasets/reanalysis-datasets/era-interim). The Gershunov et al. (2017) AR catalog are from the Scripps Institution of Oceanography (https://weclima.ucsd.edu/data-products/). Codes used to produce the results in the main text are available through Zenodo (https://zenodo.org/record/5821773). The other ARTMIP Tier 1 data sets, considered in the Supporting Information, are from the Climate Data Gateway at NCAR (https://www.earthsystemgrid.org/dataset/ucar.cgd.ccsm4.artmip.tier1.html). ARTMIP is a grass‐roots community effort and includes a collection of international researchers from universities, laboratories, and agencies. Cochairs and committee members include Jonathan Rutz, Christine Shields, L. Ruby Leung, F. Martin Ralph, and Michael Wehner, Ashley Payne, and Travis O’Brien. Details on catalogues developers can be found on the ARTMIP website. ARTMIP has received support from the US Department of Energy Office of Science Biological and Environmental Research (BER) as part of the Regional and Global Climate Modeling program, and the Center for Western Weather and Water Extremes (CW3E) at Scripps Institute for Oceanography at the University of California, San Diego.

## References

[grl63619-bib-0001] AghaKouchak, A. , Chiang, F. , Huning, L. S. , Love, C. A. , Mallakpour, I. , Mazdiyasni, O. , et al. (2020). Climate extremes and compound hazards in a warming World. Annual Review of Earth and Planetary Sciences, 48, 519–548. 10.1146/annurev-earth-071719-055228

[grl63619-bib-0002] Battisti, D. S. , & Hickey, B. M. (1984). Application of remote wind‐forced coastal trapped wave theory to the Oregon and Washington coasts. Journal of Physical Oceanography, 14, 887–903. 10.1175/1520-0485(1984)014<0887:AORWFC>2.0.CO;2

[grl63619-bib-0003] Bromirski, P. D. , Flick, R. E. , & Miller, A. J. (2017). Storm surge along the Pacific coast of North America. Journal of Geophysical Research: Oceans, 122, 441–457. 10.1002/2016JC012178

[grl63619-bib-0004] Carvajal, M. , Winckler, P. , Garreaud, R. , Igualt, F. , Contreras‐López, M. , Averil, P. , et al. (2021). Extreme sea levels at Rapa Nui (Easter Island) during intense atmospheric rivers. Natural Hazards, 106, 1619–1637. 10.1007/s11069-020-04462-2

[grl63619-bib-0005] Chapman, D. C. (1987). Application of wind‐forced, long, coastal‐trapped wave theory along the California coast. Journal of Geophysical Research, 92(C2), 1798–1816. 10.1029/JC092iC02p01798

[grl63619-bib-0006] Cordeira, J. M. , Ralph, F. M. , & Moore, B. J. (2013). The development and evolution of two atmospheric rivers in proximity to western North Pacific tropical cyclones in October 2010. Monthly Weather Review, 141, 2434–4255. 10.1175/mwr-d-13-00019.1

[grl63619-bib-0007] Dangendorf, S. , Arns, A. , Pinto, J. G. , Ludwig, P. , & Jensen, J. (2016). The exceptional influence of storm ‘Xaver’ on design water levels in the German Bight. Environmental Research Letters, 11, 054001. 10.1088/1748-9326/11/5/054001

[grl63619-bib-0008] Dee, D. P. , Uppala, S. M. , Simmons, A. J. , Berrisford, P. , Poli, P. , Kobayashi, S. , et al. (2011). The ERA‐Interim reanalysis: Configuration and performance of the data assimilation system. Quarterly Journal of the Royal Meteorological Society, 137, 553–597. 10.1002/qj.828

[grl63619-bib-0009] Dettinger, M. D. (2011). Climate change, atmospheric rivers, and floods in California—A multimodel analysis of storm frequency and magnitude changes. Journal of the American Water Resources Association, 14, 514–523. 10.1111/j.1752-1688.2011.00546.x

[grl63619-bib-0010] Dettinger, M. D. (2013). Atmospheric rivers as drought busters on the US West Coast. Journal of Hydrometeorology, 14, 1721–1732. 10.1175/jhm-d-13-02.1

[grl63619-bib-0011] Du, X. , Hendy, I. , & Schimmelmann, A. (2018). A 9000‐year flood history for southern California: A revised stratigraphy of varved sediments in Santa Barbara basin. Marine Geology, 397, 29–42. 10.1016/j.margeo.2017.11.014

[grl63619-bib-0012] Espinoza, V. , Waliser, D. E. , Guan, B. , Lavers, D. A. , & Ralph, F. M. (2018). Global analysis of climate change projection effects on atmospheric rivers. Geophysical Research Letters, 45, 4299–4308. 10.1029/2017GL076968

[grl63619-bib-0013] Gershunov, A. , Shulgina, T. , Ralph, F. M. , Lavers, D. A. , & Rutz, J. J. (2017). Assessing the climate‐scale variability of atmospheric rivers affecting western North America. Geophysical Research Letters, 44, 7900–7908. 10.1002/2017GL074175

[grl63619-bib-0014] Gill, A. E. (1982). Atmosphere‐ocean dynamics (p. 662). San Diego, CA: Academic Press.

[grl63619-bib-0015] Gill, A. E. , & Clarke, A. J. (1974). Wind‐induced upwelling, coastal currents, and sea‐level changes. Deep‐Sea Research, 21, 325–345. 10.1016/0011-7471(74)90038-2

[grl63619-bib-0016] Gupta, H. V. , Kling, H. , Yilmaz, K. K. , & Martinez, G. F. (2009). Decomposition of the mean squared error and NSE performance criteria: Implications for improving hydrological modelling. Journal of Hydrology, 377, 80–91. 10.1016/j.jhydrol.2009.08.003

[grl63619-bib-0017] Hague, B. S. , & Taylor, A. J. (2021). Tide‐only inundation: A metric to quantify the contribution of tides to coastal inundation under sea‐level rise. Natural Hazards, 107, 675–695. 10.1007/s11069-021-04600-4

[grl63619-bib-0018] Hendy, I. L. , Napier, T. J. , & Schimmelmann, A. (2015). From extreme rainfall to drought: 250 years of annually resolved sediment deposition in Santa Barbara basin, California. Quaternary International, 387, 3–12. 10.1016/j.quaint.2015.01.026

[grl63619-bib-0019] Hino, M. , Belanger, S. T. , Field, C. B. , Davis, A. R. , & Mach, K. J. (2019). High‐tide flooding disrupts local economic activity. Science Advances, 5, eaau2736. 10.1126/sciadv.aau2736 30793026 PMC6377275

[grl63619-bib-0020] Jevrejeva, S. , Frederikse, T. , Kopp, R. E. , Le Cozannet, G. , Jackson, L. P. , & van de Wal, R. S. W. (2019). Probabilistic sea level projections at the coast by 2100. Surveys in Geophysics, 40, 1673–1696. 10.1007/s10712-019-09550-y

[grl63619-bib-0021] Kalnay, E. , Kanamitsu, M. , Kistler, R. , Collins, W. , Deaven, D. , Gandin, L. , et al. (1996). The NCEP/NCAR 40‐year reanalysis project. Bulletin of the American Meteorological Society, 77(3), 437–471. 10.1175/1520-0477(1996)077<0437:TNYRP>2.0.CO;2

[grl63619-bib-0022] Khouakhi, A. , & Villarini, G. (2016). On the relationship between atmospheric rivers and high sea water levels along the US West Coast. Geophysical Research Letters, 43, 8815–8822. 10.1002/2016GL070086

[grl63619-bib-0023] Kopp, R. E. , DeConto, R. M. , Bader, D. A. , Hay, C. C. , Horton, R. M. , Kulp, S. , et al. (2017). Evolving understanding of Antarctic Ice‐Sheet physics and Ambiguity in Probabilistic sea‐level projections. Earth's Future, 5, 1217–1233. 10.1002/2017EF000663

[grl63619-bib-0024] Kriebel, D. L. , & Geiman, J. D. (2013). A coastal flood stage to define existing and future sea‐level hazards. Journal of Coastal Research, 30(5), 1017–1024.

[grl63619-bib-0025] Li, J. (2016). Assessing spatial predictive models in the environmental sciences: Accuracy measures, data variation and variance explained. Environmental Modelling & Software, 80, 1–8. 10.1016/j.envsoft.2016.02.004

[grl63619-bib-0026] Marcos, M. , Calafat, F. M. , Berihuete, A. , & Dangendorf, S. (2015). Long‐term variations in global sea level extremes. Journal of Geophysical Research: Oceans, 120, 8115–8134. 10.1002/2015JC011173

[grl63619-bib-0027] Menéndez, M. , & Woodworth, P. L. (2010). Changes in extreme high water levels based on a quasi‐global tide‐gauge data set. Journal of Geophysical Research, 115, C10011. 10.1029/2009JC005997

[grl63619-bib-0028] Moftakhari, H. R. , AghaKouchak, A. , Sanders, B. F. , Allaire, M. , & Matthew, R. A. (2018). What is nuisance flooding? Defining and monitoring an emerging challenge. Water Resources Research, 54, 4218–4227. 10.1029/2018WR022828

[grl63619-bib-0029] Moftakhari, H. R. , AghaKouchak, A. , Sanders, B. F. , & Matthew, R. A. (2017). Cumulative hazard: The case of nuisance flooding. Earth's Future, 5, 214–223. 10.1002/2016EF000494

[grl63619-bib-0030] Mudelsee, M. (2020). Statistical analysis of climate extremes (p. 200). Cambridge, UK: Cambridge University Press.

[grl63619-bib-0031] Nash, J. E. , & Sutcliffe, J. V. (1970). River flow forecasting through conceptual models. Part I—A discussion of principles. Journal of Hydrology, 10(3), 282–290. 10.1016/0022-1694(70)90255-6

[grl63619-bib-0032] Neiman, P. J. , Ralph, F. M. , Wick, G. A. , Lundquist, J. D. , & Dettinger, M. D. (2008). Meteorological characteristics and overland precipitation impacts of atmospheric rivers affecting the West Coast of North America based on eight years of SSM/I satellite observations. Journal of Hydrometeorology, 9, 22–47. 10.1175/2007jhm855.1

[grl63619-bib-0033] Newman, M. , Kiladis, G. N. , Weickmann, K. M. , Ralph, F. M. , & Sardeshmukh, P. D. (2012). Relative contributions of synoptic and low‐frequency eddies to time‐mean atmospheric moisture transport, including the role of atmospheric rivers. Journal of Climate, 25, 7341–7361. 10.1175/jcli-d-11-00665.1

[grl63619-bib-0034] Oakley, N. S. , Lancaster, J. T. , Hatchett, B. J. , Stock, J. , Ralph, F. M. , Roj, S. , & Lukashov, S. (2018). A 22‐year climatology of cool season hourly precipitation thresholds conducive to shallow landslides in California. Earth Interactions, 22, 1–35. 10.1175/ei-d-17-0029.1 31097909

[grl63619-bib-0035] Oakley, N. S. , Lancaster, J. T. , Kaplan, M. L. , & Ralph, F. M. (2017). Synoptic conditions associated with cool season post‐fire debris flows in the Transverse Ranges of southern California. Natural Hazards, 88, 327–354.

[grl63619-bib-0036] Payne, A. E. , Demory, M.‐E. , Leung, L. R. , Ramos, A. M. , Shields, C. A. , Rutz, J. J. , et al. (2020). Responses and impacts of atmospheric rivers to climate change. Nature Reviews Earth & Environment, 1, 143–157. 10.1038/s43017-020-0030-5

[grl63619-bib-0037] Pugh, D. , & Woodworth, P. (2014). Sea‐level science: Understanding tides, surges, Tsunamis, and mean sea‐level changes (p. 407). Cambridge, UK: Cambridge University Press.

[grl63619-bib-0038] Ralph, F. M. , Coleman, T. A. , Neiman, P. J. , Zamora, R. J. , & Dettinger, M. D. (2013). Observed impacts of duration and seasonality of atmospheric river landfalls on soil moisture and runoff in coastal Northern California. Journal of Hydrometeorology, 14, 443–459. 10.1175/jhm-d-12-076.1

[grl63619-bib-0039] Ralph, F. M. , Iacobellis, S. F. , Neiman, P. J. , Cordeira, J. M. , Spackman, J. R. , Waliser, D. E. , et al. (2017). Dropsonde observations of total integrated water vapor transport within North Pacific atmospheric rivers. Journal of Hydrometeorology, 18, 2577–2596. 10.1175/jhm-d-17-0036.1

[grl63619-bib-0040] Ralph, F. M. , Neiman, P. J. , & Wick, G. A. (2004). Satellite and CALJET aircraft observations of atmospheric rivers over the eastern North Pacific Ocean during the winter of 1997/98. Monthly Weather Review, 132, 1721–1745. 10.1175/1520-0493(2004)132<1721:SACAOO>2.0.CO;2

[grl63619-bib-0041] Ralph, F. M. , Wilson, A. M. , Shulgina, T. , Kawzenuk, B. , Sellars, S. , Rutz, J. J. , et al. (2019). ARTMIP‐early start comparison of Atmospheric River detection tools: How many atmospheric rivers hit northern California’s Russian river watershed? Climate Dynamics, 52, 4973–4994. 10.1007/s00382-018-4427-5

[grl63619-bib-0042] Ray, R. D. , & Merrifield, M. A. (2019). The semiannual and 4.4‐year modulations of extreme high tides. Journal of Geophysical Research: Oceans, 124, 5907–5922. 10.1029/2019JC015061

[grl63619-bib-0043] Ridder, N. , de Vries, H. , & Drijfhout, S. (2018). The role of atmospheric rivers in compound events consisting of heavy precipitation and high storm surges along the Dutch coast. Natural Hazards and Earth System Sciences, 18, 3311–3326. 10.5194/nhess-18-3311-2018

[grl63619-bib-0044] Rutz, J. J. , Shields, C. A. , Lora, J. M. , Payne, A. E. , Guan, B. , Ullrich, P. , et al. (2019). The Atmospheric River tracking method intercomparison project (ARTMIP): Quantifying uncertainties in Atmospheric River climatology. Journal of Geophysical Research: Atmospheres, 124, 13777–13802. 10.1029/2019JD030936

[grl63619-bib-0045] Ryan, H. F. , & Noble, M. A. (2006). Alongshore wind forcing of coastal sea level as a function of frequency. Journal of Physical Oceanography, 36, 2173–2184. 10.1175/jpo2972.1

[grl63619-bib-0046] Serafin, K. A. , Ruggiero, P. , & Stockdon, H. F. (2017). The relative contributions of waves, tides, and nontidal residuals to extreme total water levels on U.S. West Coast sandy beaches. Geophysical Research Letters, 44, 1839–1847. 10.1002/2016GL071020

[grl63619-bib-0047] Shepherd, T. G. (2014). Atmospheric circulation as a source of uncertainty in climate change projections. Nature Geoscience, 7, 703–708. 10.1038/ngeo2253

[grl63619-bib-0048] Shields, C. A. , Rutz, J. J. , Leung, L.‐Y. , Ralph, F. M. , Wehner, M. , Kawzenuk, B. , et al. (2018). Atmospheric River tracking method intercomparison project (ARTMIP): Project goals and experimental design. Geoscientific Model Development, 11, 2455–2474. 10.5194/gmd-11-2455-2018

[grl63619-bib-0049] Shinoda, T. , Zamudio, L. , Guo, Y. , Metzger, E. J. , & Fairall, C. W. (2019). Ocean variability and air‐sea fluxes produced by atmospheric rivers. Scientific Reports, 9, 2152. 10.1038/s41598-019-38562-2 30770858 PMC6377629

[grl63619-bib-0050] Sweet, W. V. , Dusek, G. , Obeysekera, O. , & Marra, J. (2018). Patterns and projections of high tide flooding along the U.S. Coastline using a common impact threshold (NOAA Tech. Rep. NOS CO‐OPS 086, p. 56).

[grl63619-bib-0051] Sweet, W. V. , & Park, J. (2014). From the extreme to the mean: Acceleration and tipping points of coastal inundation from sea level rise. Earth's Future, 2, 579–600. 10.1002/2014EF000272

[grl63619-bib-0052] Sweet, W. V. , Simon, S. , Dusek, G. , Marcy, D. , Brooks, W. , Pendleton, M. , & Marra, J. (2021). 2021 State of high tide flooding and annual Outlook (p. 28). National Oceanic and Atmospheric Administration.

[grl63619-bib-0053] Theuerkauf, E. J. , Rodriguez, A. B. , Fegley, S. R. , & Luettich, R. A. (2014). Sea level anomalies exacerbate beach erosion. Geophysical Research Letters, 41, 5139–5147. 10.1002/2014GL060544

[grl63619-bib-0054] Thompson, P. R. , Widlansky, M. J. , Hamlington, B. D. , Merrifield, M. A. , Marra, J. J. , Mitchum, G. T. , & Sweet, W. (2021). Rapid increases and extreme months in projections of United States high‐tide flooding. Nature Climate Change, 11, 584–590. 10.1038/s41558-021-01077-8

[grl63619-bib-0055] Thomson, R. E. , & Emery, W. J. (2014). Data analysis methods in physical Oceanography (3rd ed., p. 716). Elsevier.

[grl63619-bib-0056] Vallis, G. K. , Zurita‐Gotor, P. , Cairns, C. , & Kidston, J. (2015). Response of the large‐scale structure of the atmosphere to global warming. Quarterly Journal of the Royal Meteorological Society, 141, 1479–1501. 10.1002/qj.2456

[grl63619-bib-0057] Verdy, A. , Mazloff, M. R. , Cornuelle, B. D. , & Kim, S. Y. (2014). Wind‐driven sea level variability on the California coast: An adjoint sensitivity analysis. Journal of Physical Oceanography, 44, 297–318. 10.1175/jpo-d-13-018.1

[grl63619-bib-0058] Wang, S. Y. S. , Yoon, J.‐H. , Becker, E. , & Gillies, R. (2017). California from drought to deluge. Nature Climate Change, 7, 465–468. 10.1038/nclimate3330

[grl63619-bib-0059] White, A. B. , Moore, B. J. , Gottas, D. J. , & Neiman, P. J. (2019). Winter storm conditions leading to excessive runoff above California’s Oroville Dam during January and February 2017. Bulletin of the American Meteorological Society, 100, 55–70. 10.1175/bams-d-18-0091.1

[grl63619-bib-0060] Yin, J. H. (2005). A consistent poleward shift of the storm tracks in simulations of 21st century climate. Geophysical Research Letters, 32, L18701. 10.1029/2005GL023684

[grl63619-bib-0061] Zhu, Y. , & Newell, R. E. (1998). A proposed algorithm for moisture fluxes from atmospheric rivers. Monthly Weather Review, 126, 725–735. 10.1175/1520-0493(1998)126<0725:APAFMF>2.0.CO;2

[grl63619-bib-0062] Brands, S. , Gutiérrez, J. M. , & San Mart, D. (2017). Twentieth‐century Atmospheric River activity along the west coasts of Europe and north America: Algorithm formulation, reanalysis uncertainty and links to atmospheric circulation patterns. Climate Dynamics, 48(9–10), 2771–2795.

[grl63619-bib-0063] Efron, B. , & Hastie, T. (2016). Computer age statistical inference: Algorithms, evidence, and data science (p. 475). Cambridge, UK: Cambridge University Press.

[grl63619-bib-0064] Gelaro, R. , McCarty, W. , Suárez, M. J. , Todling, R. , Molod, A. , Takacs, L. , et al. (2017). The Modern‐Era retrospective analysis for research and applications, version 2 (MERRA‐2). Journal of Climate, 30(14), 5419–5454. 32020988 10.1175/JCLI-D-16-0758.1 PMC6999672

[grl63619-bib-0065] Goldenson, N. , Leung, L. R. , Bitz, C. M. , & Blanchard‐Wrigglesworth, E. (2018). Influence of Atmospheric River events on mountain Snowpack of the western U.S. Journal of Climate, 31(24), 9921–9940.

[grl63619-bib-0066] Hagos, S. , Leung, L. R. , Yang, Q. , Zhao, C. , & Lu, J. (2015). Resolution and dynamical core dependence of atmospheric river frequency in global model simulations. Journal of Climate, 28(7), 2764–2776.

[grl63619-bib-0067] Leung, L. R. , & Qian, Y. (2009). Atmospheric rivers induced heavy precipitation and flooding in the western U.S. simulated by the WRF regional climate model. Geophysical Research Letters, 36, L03820. 10.1029/2008GL036445

[grl63619-bib-0068] Lora, J. M. , Mitchell, J. L. , Risi, C. , & Tripati, A. E. (2017). North Pacific atmospheric rivers and their influence on western north America at the Last Glacial maximum. Geophysical Research Letters, 44, 1051–1059. 10.1002/2016GL071541

[grl63619-bib-0069] McClenny, E. E. , Ullrich, P. A. , & Grotjahn, R. (2020). Sensitivity of atmospheric river vapor transport and precipitation to uniform sea surface temperature increases. Journal of Geophysical Research: Atmospheres, 125, e2020JD033421. 10.1029/2020JD033421 PMC7771035 33391965

[grl63619-bib-0070] Mundhenk, B. D. , Barnes, E. A. , & Maloney, E. D. (2016). All‐season climatology and variability of atmospheric river frequencies over the North Pacific. Journal of Climate, 29(13), 4885–4903.

[grl63619-bib-0071] Muszynski, G. , Kashinath, K. , Kurlin, V. , & Wehner, M. (2019). Topological data analysis and machine learning for recognizing atmospheric river patterns in large climate datasets. Geoscientific Model Development, 12(2), 613–628. 10.1029/2020JD033421

[grl63619-bib-0072] Pan, M. , & Lu, M. (2019). A novel atmospheric river identification algorithm. Water Resources Research, 55, 6069–6087. 10.1029/2018WR024407

[grl63619-bib-0073] Ponte, R. M. (2006). Oceanic response to surface loading effects neglected in volume‐conserving models. Journal of Physical Oceanography, 36, 426–434. 10.1175/JPO2843.1

[grl63619-bib-0074] PrabhatKashinath, K. , Mudigonda, M. , Kim, S. , Kapp‐Schwoerer, L. , Graubner, A. , Karaismailoglu, E. , et al. (2021). ClimateNet: An expert‐labeled open dataset and deep learning architecture for enabling high‐precision analyses of extreme weather. Geoscientific Model Development, 14, 107–124.

[grl63619-bib-0075] Reid, K. J. , King, A. D. , Lane, T. P. , & Short, E. (2020). The sensitivity of Atmospheric River Identification to integrated water vapor transport threshold, resolution, and regridding method. Journal of Geophysical Research: Atmospheres, 125, e2020JD032897. 10.1029/2020JD032897

[grl63619-bib-0076] Rhoades, A. , Jones, A. , O’Brien, T. A. , O’Brien, J. P. , Ullrich, P. A. , & Zarzycki, C. M. (2020). Influences of North Pacific Ocean domain extent on the western US winter hydroclimatology in variable‐resolution CESM. Journal of Geophysical Research: Atmospheres, 125, e2019JD031977. 10.1029/2019JD031977

[grl63619-bib-0077] Rutz, J. J. , Steenburgh, W. J. , & Ralph, F. M. (2014). Climatological characteristics of atmospheric rivers and their inland penetration over the western United States. Monthly Weather Review, 142, 905–920.

[grl63619-bib-0078] Shearer, E. J. , Nguyen, P. , Sellars, S. L. , Analui, B. , Kawzenuk, B. , Hsu, K. , & Sorooshian, S. (2020). Examination of global midlatitude atmospheric river lifecycles using an object?oriented methodology. Journal of Geophysical Research: Atmospheres, 125, e2020JD033425. 10.1029/2020JD033425

[grl63619-bib-0079] Shields, C. A. , & Kiehl, J. T. (2016a). Atmospheric river landfall‐latitude changes in future climate simulations. Geophysical Research Letters, 43, 8775–8782. 10.1002/2016GL070470

[grl63619-bib-0080] Shields, C. A. , & Kiehl, J. T. (2016b). Simulating the pineapple express in the half degree community climate system model, CCSM4. Geophysical Research Letters, 43, 7767–7773. 10.1002/2016GL069476

[grl63619-bib-0081] Skinner, C. B. , Lora, J. M. , Payne, A. E. , & Poulsen, C. J. (2020). Atmospheric river changes shaped mid‐latitude hydroclimate since the mid‐Holocene. Earth and Planetary Science Letters, 541, 116293.

[grl63619-bib-0082] Ullrich, P. A. , & Zarzycki, C. M. (2017). TempestExtremes: A framework for scale‐insensitive pointwise feature tracking on unstructured grids. Geoscientific Model Development, 10, 1069–1090.

[grl63619-bib-0083] Xu, G. , Ma, X. , Chang, P. , & Wang, L. (2020). Image‐processing‐based atmospheric river tracking method version 1 (IPART‐1). Geoscientific Model Development, 13, 4639–4662.

