# Supplementary material for: High‐Tide Floods and Storm Surges During Atmospheric Rivers on the US West Coast
Source: Geophys Res Lett. 2022 Jan 25;49(2):e2021GL096820. doi: 10.1029/2021GL096820 (PMC9539697; doi:10.1029/2021GL096820)
Supplement: Supplementary file 1 — Supporting Information S1 [file GRL-49-e2021GL096820-s001.pdf]

Supporting Information for "High-Tide Floods and Storm Surges During Atmospheric Rivers on the US West Coast"

Christopher G. Piecuch[1], Sloan Coats[2], Sönke Dangendorf[3], Felix W. Landerer[4], J. T. Reager[4], Philip R. Thompson[2], and Thomas Wahl[5]

Christopher G. Piecuch, Woods Hole Oceanographic Institution, 266 Woods Hole Road, Woods Hole, MA 02543, USA. (cpiecuch@whoi.edu)

[1]Woods Hole Oceanographic Institution, Woods Hole, MA, USA.

[2]University of Hawai'i at Mānoa, Honolulu, HI, USA.

[3]Old Dominion University, Norfolk, VA, USA

[4]Jet Propulsion Laboratory, California Institute of Technology, Pasadena, CA, USA.

[5]University of Central Florida, Orlando, FL, USA.

Contents of this file

1. Text S1 to S5
2. Figures S1 to S7
3. Tables S1 to S3

S1. Bootstrapping

We use bootstrapping to quantify uncertainty related to the finite record lengths of the data (e.g., Efron and Hastie, 2016). Given time-series data (e.g., hourly tide-gauge water-level observations), for each sample statistic (e.g., mean, standard deviation), we perform 1,000 iterations of randomly selecting (with replacement) a number of data values equal to the length of the original data record and computing the sample statistic. Since values can be repeated or omitted, statistics computed during any given iteration can differ from the value computed from the original data. Values in the main text are usually given in the form of averages or 95% confidence intervals from the resulting distributions.

Note that, for quantities that depend on the covariance between time series (e.g., variance explained, co-occurrence of HTFs and ARs), we randomly select the time points at each bootstrapping iteration and use those common time points for each data series involved in the calculation. For example, we compute regression coefficients using contemporaneous storm surge, wind stress, barometric pressure, and freshwater flux.

A caveat of the bootstrapping method used here is that it is performed independently at each tide-gauge location. Thus, when computing spatial averages, we will tend to underestimate the true uncertainties, since the approach effectively assumes that errors

are uncorrelated across tide gauges. In reality, there are spatial dependencies in the processes under consideration that should be taken into account in a more complete future spatiotemporal statistical analysis.

S2. Hypothesis testing

To evaluate whether relationships between quantities of interest in section 3 of the main text are statistically significant, we run Monte Carlo simulations of synthetic stochastic processes. For example, we compute the significance of the co-occurrence of (or correlation between) HTFs and ARs by comparing observed values (Figures 2, 3) to values expected from two independent stochastic daily Poisson processes with parameter values determined from the observed numbers of HTF days and AR days during the study period. The corresponding P-value is calculated as the fraction of the time that co-occurrences are more frequent (or that correlations are stronger) in the simulations than in the observations. Likewise, we quantify the significance of the correlation between interannual time series of HTFs and mean sea level (Figure 3b) by comparing to simulated correlations between a random Poisson process with parameter value based on the observed number of HTFs and a random zero-mean Gaussian process with variance parameter equal to the variance of the observed mean sea-level time series.

S3. Ridge regression

Consider the linear model

$$\mathbf{y} = \mathbf{X}\beta + \epsilon \tag{S1}$$

where $\mathbf{y}$ is the $n \times 1$ known observational vector, $\mathbf{X}$ is the $n \times p$ known structure matrix, ϵ is the $n \times 1$ noise vector, and β is the $p \times 1$ vector of unknown parameters to be determined. With reference to Eq. (1) in the main text, the vector $\mathbf{y}$ in Eq. (S1) corresponds to the observed storm surge, matrix $\mathbf{X}$ corresponds to the local wind, pressure, and precipitation forcing, and vector β corresponds to the a and b terms.

The ordinary least squares estimate of the parameter vector is

$$\hat{\beta}_{\mathrm{OLS}} = \left(\mathbf{X}^{\mathsf{T}}\mathbf{X}\right)^{-1}\mathbf{X}^{\mathsf{T}}\mathbf{y}. \tag{S2}$$

If elements of the structure matrix are collinear, then the inner product matrix $\mathbf{X}^{\mathsf{T}}\mathbf{X}$ can be poorly conditioned (or even singular), resulting in large uncertainties on $\hat{\beta}_{\mathrm{OLS}}$. This is a concern in the present context, since the predictor variables can be correlated. As just one randomly selected example, the Pearson correlation coefficient between anomalous meridional wind stress and barometric pressure across all landfalling ARs at Port Chicago, California during 1980–2016 is -0.53 ($P < 0.01$).

Ridge regression is a regularization technique that gives more accurate (but biased) estimates relative to ordinary least squares in problems with correlated predictors. The ridge-regression estimate of the parameter vector is (e.g., Efron and Hastie, 2016)

$$\hat{\beta}_{\mathrm{RR}} = \left(\mathbf{X}^{\mathsf{T}}\mathbf{X} + \lambda\mathsf{I}\right)^{-1}\mathbf{X}^{\mathsf{T}}\mathbf{y}. \tag{S3}$$

where $\lambda > 0$ is a real constant and I is the identity matrix. See Efron and Hastie (2016) for a Bayesian interpretation of λ in terms of prior belief.

We use Eq. (S3) with $\lambda = 0.3$ to solve for the a's and b's in Eq. (1) in the main text. Results are robust to the selection of λ, and similar regression coefficients are found for a wide range of λ values (Figure S4). Before evaluating Eq. (S3), we standardize the

predictors to have zero mean and unit sum of squares. We also remove the mean from the observational vector. After computing $\hat{\beta}_{\mathrm{RR}}$, we rescale the regression coefficients back to their respective physical units (cf. Figures S4, S5).

S4. Theoretical coefficients

To interpret regression coefficients determined empirically from the data (Figures S4, S5), we build a model of the coastal sea-level response to surface wind, pressure, and precipitation forcing. Imagine a straight coastline extending infinitely in the meridional/alongshore (y) coordinate. The coast faces the ocean to the west, with the origin in the zonal/onshore coordinate (x) at the coast. Offshore positions have values $x < 0$. We consider the following form of shallow water equations

$$\eta_t + H u_x = 0, \tag{S4}$$

$$-fv = -g \left[\eta + \frac{1}{\rho g} p + \int^t q(t')\ dt' \right]_x + \frac{1}{\rho H} \pi, \tag{S5}$$

$$v_t + fu = \frac{1}{\rho H} \tau - \gamma v. \tag{S6}$$

Here t is time, subscript is partial differentiation, p is barometric pressure, q is precipitation, π and τ are onshore and alongshore wind stress, respectively, η is adjusted sea level (Gill, 1982; Ponte, 2006)

$$\eta \doteq \zeta - \int^t q(t')\ dt', \tag{S7}$$

where ζ is sea level, u is onshore velocity, v is alongshore velocity, ρ is constant ocean density, g is gravitational acceleration, f is the Coriolis parameter, H is constant ocean depth, and $\gamma \doteq r/H$ is an inverse timescale, where r is a linear friction coefficient.

The choice of the locally forced form of Eqs. (S4)–(S6) is partly motivated by the regression analysis, which suggests that observed storm surges can be largely understood in terms of local wind, pressure, and precipitation forcing (Figure 5). We have omitted terms involving the onshore velocity in the onshore momentum equation, and the effects of stratification, nonlinearities, and alongshore dependence in the governing equations. These omissions follow formally from the assumptions that Burger and Rossby numbers are small, alongshore scales are much larger than onshore scales, alongshore motions are much stronger than onshore motions, and frequencies are sub-inertial.

We suppose that surface forcing by an AR is described by temporal plane waves that decay spatially away from the coast

$$F\left(x,t\right)=F_0\exp\left(kx-i\sigma t\right),\ F\in\left\{p,q,\pi,\tau\right\}, \tag{S8}$$

where $i\doteq\sqrt{-1}$, σ is angular frequency, and k and F_0 are real constants. We demand that the oceanic response is separable and described by plane waves in time

$$y\left(x,t\right)=\tilde{y}\left(x\right)\exp\left(-i\sigma t\right),\ y\in\left\{\eta,u,v\right\}, \tag{S9}$$

where $\tilde{\eta}$, $\tilde{u}$, and $\tilde{v}$ are functions of the onshore coordinate to be determined.

Inserting (S8) and (S9) into (S4)–(S6) and rearranging gives a second-order inhomogeneous linear ordinary differential equation for onshore structure

$$\tilde{\eta}_{xx}-\kappa^2\tilde{\eta}=\left[-\frac{k}{\rho g}p_0-i\frac{k}{\sigma}q_0+\frac{1}{\rho gH}\pi_0+\frac{f}{\rho gH}\left(\frac{\gamma+i\sigma}{\gamma^2+\sigma^2}\right)\tau_0\right]k\exp\left(kx\right) \tag{S10}$$

where $\kappa\doteq s\exp\left(i\varphi\right)/L_R$ is complex, with barotropic Rossby radius of deformation $L_R\doteq\sqrt{gH}/f$, amplitude $s\doteq\left[1+\left(\gamma/\sigma\right)^2\right]^{-1/4}$, and phase $\varphi\doteq\frac{1}{2}\arctan\left(-\gamma/\sigma\right)$.

The boundary conditions are

$$\eta \to 0 \text{ as } x \to -\infty, \tag{S11}$$

$$\tilde{\eta}_x = -\frac{k}{\rho g}p_0 - i\frac{k}{\sigma}q_0 + \frac{1}{\rho g H}\pi_0 + \frac{f}{\rho g H}\left(\frac{\gamma + i\sigma}{\gamma^2 + \sigma^2}\right)\tau_0 \text{ at } x = 0. \tag{S12}$$

The first boundary condition demands a shore-trapped solution, whereas the second boundary condition can be shown to be a form of no-normal flow through the boundary.

The solution to Eq. (S10) subject to Eqs. (S11) and (S12) is

$$\tilde{\eta}\left(x\right) = \frac{k\exp\left(kx\right) + \kappa\exp\left(-\kappa x\right)}{k^2 - \kappa^2}\left[-\frac{k}{\rho g}p_0 - i\frac{k}{\sigma}q_0 + \frac{1}{\rho g H}\pi_0 + \frac{f}{\rho g H}\left(\frac{\gamma + i\sigma}{\gamma^2 + \sigma^2}\right)\tau_0\right]. \tag{S13}$$

which, at the coast, simplifies to

$$\tilde{\eta}\left(x = 0\right) = \frac{1}{k - \kappa}\left[-\frac{k}{\rho g}p_0 - i\frac{k}{\sigma}q_0 + \frac{1}{\rho g H}\pi_0 + \frac{f}{\rho g H}\left(\frac{\gamma + i\sigma}{\gamma^2 + \sigma^2}\right)\tau_0\right]. \tag{S14}$$

Adding iq_0/σ to convert from effective sea level to sea level [cf. Eq. (S7)] and scaling by $\exp\left(-i\sigma t\right)$, we obtain the time-variable coastal sea-level solution

$$\zeta\left(x = 0, t\right) = \frac{1}{k - \kappa}\left[-\frac{k}{\rho g}p - i\frac{\kappa}{\sigma}q + \frac{1}{\rho g H}\pi + \frac{f}{\rho g H}\left(\frac{\gamma + i\sigma}{\gamma^2 + \sigma^2}\right)\tau\right], \tag{S15}$$

where, on the right side, we understand the forcing terms to be evaluated at the coast.

Recognizing that $i\exp\left(-i\sigma t\right) = \mathcal{H}\left[\exp\left(-i\sigma t\right)\right]$ by definition of the Hilbert transform $\mathcal{H}$, and in analogy with Eq. (1) in the main text, we can write Eq. (S15) equivalently as

$$\zeta\left(x = 0, t\right) = a_\pi \pi + b_\pi \mathcal{H}\left(\pi\right) + a_\tau \tau + b_\tau \mathcal{H}\left(\tau\right) + a_p p + b_p \mathcal{H}\left(p\right) + a_q q + b_q \mathcal{H}\left(q\right), \tag{S16}$$

where

$$a_\pi \doteq \Re\left[\frac{1}{k - \kappa}\left(\frac{1}{\rho g H}\right)\right],\ b_\pi \doteq \Im\left[\frac{1}{k - \kappa}\left(\frac{1}{\rho g H}\right)\right], \tag{S17}$$

$$a_\tau \doteq \Re\left\{\frac{1}{k - \kappa}\left[\frac{f}{\rho g H}\left(\frac{\gamma + i\sigma}{\gamma^2 + \sigma^2}\right)\right]\right\},\ b_\tau \doteq \Im\left\{\frac{1}{k - \kappa}\left[\frac{f}{\rho g H}\left(\frac{\gamma + i\sigma}{\gamma^2 + \sigma^2}\right)\right]\right\}, \tag{S18}$$

$$a_p \doteq \Re\left[\frac{1}{k-\kappa}\left(-\frac{k}{\rho g}\right)\right],\ b_p \doteq \Im\left[\frac{1}{k-\kappa}\left(-\frac{k}{\rho g}\right)\right], \tag{S19}$$

$$a_q \doteq \Re\left[\frac{1}{k-\kappa}\left(-i\frac{\kappa}{\sigma}\right)\right],\ b_q \doteq \Im\left[\frac{1}{k-\kappa}\left(-i\frac{\kappa}{\sigma}\right)\right], \tag{S20}$$

and where $\Re$ and $\Im$ correspond to real and imaginary parts, respectively.

To evaluate Eqs. (S17)–(S20), we use reasonable, representative numerical values or ranges for the various parameters (Table S2). We assume that σ is between $2\pi/(1\text{ day})$ and $2\pi/(6\text{ days})$. This range is selected because roughly two-thirds of the landfalling ARs considered here have lifetimes between 1 and 6 days (not shown).

In Figure S4, we compare numerical values of the various a and b terms determined empirically from ridge regression applied to the data to those values expected theoretically from first principles as embodied in Eqs. (S17)–(S20) and evaluated as described in the previous paragraph. Empirical values are shown as a function of ridge-regression parameter λ and represent 95% confidence intervals across all tide gauges and bootstrap iterations. Theoretical values are shown as minima and maxima based on the parameter values in Table S2 and the target frequency range.

Acknowledging that uncertainties are large, we find that empirical and theoretical coefficients are roughly consistent to order of magnitude, overlapping within their estimated uncertainties (Figure S4). This supports the hypothesis that statistical results in the main text are informative of causal relationships. Note that, in mentioning the rough consistency between empirical and theoretical results, we are not arguing that the analytical model represents all of the relevant physics underlying ζ during ARs. This model framework is highly simplified, and omits many factors that may be important in the real world (e.g., stratification, nonlinearities, alongshore dependence, topographic

variation). Our goal here was to identify a simple model based on reasonable assumptions and amenable to analytical solution to show that statistical relationships between forcing and response obtained through regression analysis are not in gross conflict with expectations from basic physics. While we believe we have largely accomplished this goal, we recognize that our results identify open questions. For example, while the estimates feature overlapping uncertainties, empirical values of a_π are largely negative, whereas first principles predict a positive a_π value (Figure S4 top left). (Keep in mind that, according to regression analysis, π is not an important ζ driver.) We speculate that this discrepancy could reflect unphysical relationships inferred by the regression analysis or important physics not represented in the analytical model. Future studies based on more comprehensive causal frameworks (e.g., high-resolution general circulation models) could revisit these questions to identify more unambiguously the relative roles of different forcing mechanisms and the nature of the oceanic response.

S5. Comparison to other AR catalogs

Our main analysis is based on the Gershunov et al. (2017) AR catalog. Results may thus be sensitive to choices made by those authors in designing their algorithm to detect AR events. As a preliminary uncertainty quantification to assess the robustness of our findings, we also considered 22 other AR catalogs for the US West Coast from ARTMIP (Table S3). These catalogs are based on different tracking algorithms applied to the Modern-Era Retrospective analysis for Research and Applications Version 2 (MERRA2) with 3-hourly time resolution and $5/8° \times 1/2°$ horizontal resolution (Gelaro et al., 2017).

Data represent Tier 1 fields given as binary indicator maps of the presence or absence of an AR during 1980–2016.

For each ARTMIP catalog, we recomputed the percentages of HTF days that are AR days and AR days that are HTF days (Figure S7). The spatial pattern of the percentage of AR days that are HTF days obtained from Gershunov et al. is very similar to the one determined from ARTMIP (Figure S7b). This means that this statistic is insensitive to choice of AR catalog. For the percentage of HTF days that are AR days, ARTMIP and Gershunov et al. give similar central values for southern and central California, but Gershunov furnishes slightly lower best estimates along northern California, Oregon, and Washington compared to ARTMIP on average (Figure S7a). The reason could be that the Gershunov et al. catalog returns fewer ARs: while the Gershunov et al. product recognizes only the landfalling location of an AR, the ARTMIP datasets identify all grid cells experiencing an AR. This interpretation is consistent with past studies reporting that AR tracking methods based on more selective criteria identify fewer AR events (Ralph et al., 2019). In any case, overall spatial patterns are broadly similar, and the Gershunov et al. estimates are everywhere enveloped by the 66% confidence intervals determined from ARTMIP. Thus, we conclude that statistics related to co-occurrences of HTFs and ARs are qualitatively robust in terms of lowest-order structure, and that results based on the Gershunov et al. dataset are broadly representative.

References

Brands, S., Gutiérrez, J. M., & San Martn, D. (2017). Twentieth-century atmospheric river activity along the west coasts of Europe and North America: algorithm formulation,

reanalysis uncertainty and links to atmospheric circulation patterns. *Climate Dynamics*, *48*, 9–10, 2771–2795.

Efron, B., & Hastie, T. (2016). Computer Age Statistical Inference: Algorithms, Evidence, and Data Science. Cambridge University Press, Cambridge, 475 pp.

Gelaro, R., McCarty, W., Suárez, M. J., Todling, R., Molod, A., Takacs, L., Randles, C. A., Darmenov, A., Bosilovich, M. G., Reichle, R., Wargan, K., Coy, L., Cullather, R., Draper, C., Akella, S., Buchard, V., Conaty, A., da Silva, A. M., Gu, W., Kim, G.-K., Koster, R., Lucchesi, R., Merkova, D., Nielsen, J. E., Partyka, Pawson, S., Putman, W., Rienecker, M., Schubert, S. D., Sienkiewicz, M., & Zhao, B. (2017). The Modern-Era Retrospective Analysis for Research and Applications, Version 2 (MERRA-2). *Journal of Climate*, *30*, 14, 5419–5454.

Gershunov, A., Shulgina, T., Ralph, F. M., Lavers, D. A., & Rutz, J. J. (2017). Assessing the climate-scale variability of atmospheric rivers affecting western North America. *Geophysical Research Letters*, *44*, 7900–7908.

Gill, A. E. (1982). Atmosphere-Ocean Dynamics. Academic Press, San Diego, 662 pp.

Goldenson, N., Leung, L. R., Bitz, C. M., & Blanchard-Wrigglesworth, E. (2018). Influence of Atmospheric River Events on Mountain Snowpack of the Western U.S. *Journal of Climate*, *31*, 24, 9921–9940.

Hagos, S., Leung, L. R., Yang, Q., Zhao, C., & Lu, J. (2015). Resolution and Dynamical Core Dependence of Atmospheric River Frequency in Global Model Simulations. *Journal of Climate*, *28*, 7, 2764–2776.

Leung, L. R., & Qian, Y. (2009). Atmospheric rivers induced heavy precipitation and flooding in the western U.S. simulated by the WRF regional climate model. *Geophysical Research*

Letters, *36*, L03820.

Lora, J. M., Mitchell, J. L., Risi, C., & Tripati, A. E. (2017). North Pacific atmospheric rivers and their influence on western North America at the Last Glacial Maximum. *Geophysical Research Letters*, *44*, 1051–1059.

McClenny, E. E., Ullrich, P. A., & Grotjahn, R. (2020). Sensitivity of atmospheric river vapor transport and precipitation to uniform sea surface temperature increases. *Journal of Geophysical Research: Atmospheres*, *125*, 21, e2020JD033421.

Mundhenk, B. D., Barnes, E. A., & Maloney, E. D. (2016). All-season climatology and variability of atmospheric river frequencies over the North Pacific. *Journal of Climate*, *29*, 13, 4885–4903.

Muszynski, G., Kashinath, K., Kurlin, V., & Wehner, M. (2019). Topological data analysis and machine learning for recognizing atmospheric river patterns in large climate datasets. *Geoscientific Model Development*, *12*, 2, 613–628.

Pan, M. & Lu, M. (2019). A Novel Atmospheric River Identification Algorithm. *Water Resources Research*, *55*, 6069–6087.

Ponte, R. M. (2006). Oceanic Response to Surface Loading Effects Neglected in Volume-Conserving Models. *Journal of Physical Oceanography*, *36*, 426–434, https://doi.org/10.1175/JPO2843.1.

Prabhat, Kashinath, K., Mudigonda, M., Kim, S., Kapp-Schwoerer, L., Graubner, A., Karaismailoglu, E., von Kleist, L., Kurth, T., Greiner, A., Mahesh, A., Yang, K., Lewis, C., Chen, J., Lou, A., Chandran, S., Toms, B., Chapman, W., Dagon, K., Shields, C. A., O'Brien, T., Wehner, M,. & Collins, W. (2021). ClimateNet: an expert-labeled open dataset

and deep learning architecture for enabling high-precision analyses of extreme weather. *Geoscientific Model Development*, *14*, 107–124.

Ralph, F. M., Wilson, A. M., Shulgina, T., Kawzenuk, B., Sellars, S., Rutz, J. J., Lamjiri, M. A., Barnes, E. A., Gershunov, A., Guan, B., Nardi, K. M., Osborne, T., & Wick, G. A. (2019). ARTMIP-early start comparison of atmospheric river detection tools: how many atmospheric rivers hit northern California's Russian River watershed? *Climate Dynamics*, *52*, 4973–4994.

Reid, K. J., King, A. D., Lane, T. P., & Short, E. (2020). The Sensitivity of Atmospheric River Identification to Integrated Water Vapor Transport Threshold, Resolution, and Regridding Method. *Journal of Geophysical Research: Atmospheres*, *125*, 20, e2020JD032897.

Rhoades, A., Jones, A., O'Brien, T. A., O'Brien, J. P., Ullrich, P. A., Zarzycki, C. M (2020). Influences of North Pacific Ocean domain extent on the western US winter hydroclimatology in variable-resolution CESM. *Journal of Geophysical Research: Atmospheres*, *125*, 14, e2019JD031977.

Rutz, J. J., Steenburgh, W. J., & Ralph, F. M. (2014). Climatological characteristics of atmospheric rivers and their inland penetration over the western United States. *Monthly Weather Review*, *142*, 905–920.

Shearer, E. J., Nguyen, P., Sellars, S. L., Analui, B., Kawzenuk, B., Hsu, K., & Sorooshian, S. (2020). Examination of global midlatitude atmospheric river lifecycles using an object?oriented methodology. *Journal of Geophysical Research: Atmospheres*, *125*, 22, e2020JD033425.

Shields, C. A., & Kiehl, J. T. (2016a). Atmospheric river landfall-latitude changes in future climate simulations. *Geophysical Research Letters*, *43*, 8775–8782.

Shields, C. A., & Kiehl, J. T. (2016b). Simulating the Pineapple Express in the half degree Community Climate System Model, CCSM4. *Geophysical Research Letters*, *43*, 7767–7773.

Skinner, C. B., Lora, J. M., Payne, A. E., & Poulsen, C. J. (2020). Atmospheric river changes shaped mid-latitude hydroclimate since the mid-Holocene. *Earth and Planetary Science Letters*, *541*, 116293.

Ullrich, P. A., & Zarzycki, C. M. (2017). TempestExtremes: a framework for scale-insensitive pointwise feature tracking on unstructured grids. *Geoscientific Model Develompent*, *10*, 1069–1090.

Xu, G., Ma, X., Chang, P., & Wang, L. (2020). Image-processing-based atmospheric river tracking method version 1 (IPART-1). *Geoscientific Model Develompent*, *13*, 4639–4662.

Station	ID	Latitude	Longitude	Completeness	Threshold (cm)	99.5th Percentile (cm)
San Diego	9410170	32.7°N	117.2°W	99.2%	57.0	37.8
La Jolla	9410230	32.9°N	117.3°W	99.7%	56.5	36.3
Los Angeles	9410660	33.7°N	118.3°W	100.0%	56.7	36.0
Santa Monica	9410840	34°N	118.5°W	91.4%	56.6	37.0
Port San Luis	9412110	35.2°N	120.8°W	99.5%	56.5	32.4
Monterey	9413450	36.6°N	121.9°W	99.7%	56.5	31.7
Alameda	9414750	37.8°N	122.3°W	99.8%	58.0	27.4
San Francisco	9414290	37.8°N	122.5°W	99.8%	57.1	28.1
Point Reyes	9415020	38.0°N	123.0°W	98.9%	57.0	32.5
Port Chicago	9415144	38.1°N	122.0°W	98.5%	56.0	26.9
Arena Cove	9416841	38.9°N	123.7°W	78.8%	57.2	34.8
Humboldt Bay	9418767	40.8°N	124.2°W	98.4%	58.4	37.7
Crescent City	9419750	41.7°N	124.2°W	98.8%	58.4	36.0
Port Orford	9431647	42.7°N	124.5°W	87.2%	58.9	39.3
Charleston	9432780	43.3°N	124.3°W	98.5%	59.3	40.5
South Beach	9435380	44.6°N	124.0°W	99.3%	60.2	43.3
Astoria	9439040	46.2°N	123.8°W	99.3%	60.5	44.4
Toke Point	9440910	46.7°N	124.0°W	92.2%	60.9	51.1
Seattle	9447130	47.6°N	122.3°W	100.0%	63.8	34.9
Port Townsend	9444900	48.1°N	122.8°W	99.6%	60.4	36.3
Port Angeles	9444090	48.1°N	123.4°W	98.8%	58.6	41.5
Neah Bay	9443090	48.4°N	124.6°W	99.7%	59.7	46.0
Friday Harbor	9449880	48.5°N	123.0°W	99.9%	59.5	39.1
Cherry Point	9449424	48.9°N	122.8°W	98.5%	61.2	37.2

Table S1. Name, identification number, latitude, longitude, completeness, HTF threshold, and 99.5th percentile of tide-gauge stations and their hourly still water level records during 1980–2016. Identification numbers are as provided by NOAA. Completeness refers to the percentage of hours during the study period for which the tide gauge returned valid hourly still water level data. HTF threshold is a linear function of great diurnal range (difference between mean higher high water and mean lower low water) after Sweet et al. (2018). Values for HTF threshold and 99.5th percentile are relative to mean higher high water. Note that the Humboldt Bay tide gauge is also known as North Spit.

Parameter	Description	Value
ζ	Sea Level	—
η	Effective Sea Level	—
u	Onshore Velocity	—
v	Alongshore Velocity	—
τ	Meridional Wind Stress	—
π	Zonal Wind Stress	—
q	Precipitation	—
p	Barometric Pressure	—
t	Time	—
x	Onshore Coordinate	—
σ	Angular Frequency	—
ρ	Ocean Density	1000 kg m^{-3}
g	Gravitational Acceleration	10 m s^{-2}
k	Offshore Decay Scale	50–200 km
H	Shelf Depth	100–200 m
f	Coriolis Parameter	0.6–1.1$\times 10^{-4}$ s^{-1}
r	Friction Coefficient	1×10^{-4}–1×10^{-2} m s^{-1}
γ	Inverse Frictional Timescale	5×10^{-7}–1×10^{-4} s^{-1}

Table S2. Analytical model variables and parameters. Reasonable parameter values and ranges are given where applicable.

No	Dataset	Reference
1	ar-connect	Sellars et al. (2015); Shearer et al. (2020)
2	brands_v1	Brands et al. (2017)
3	brands_v2	Brands et al. (2017)
4	brands_v3	Brands et al. (2017)
5	ClimateNet_DL	Prabhat et al. (2020)
6	goldenson_v1-1	Goldenson et al. (2018)
7	goldenson_v1	Goldenson et al. (2018)
8	IPART	Xu et al. (2020)
9	lbnl_ML_TDA	Muszynski et al. (2019)
10	lora_v2_global	Lora et al. (2017)
11	mundhenk_v2	Mundhenk et al. (2016)
12	mundhenk_v3	Mundhenk et al. (2016)
13	PanLu	Pan and Lu (2019)
14	pnnl1_hagos	Hagos et al. (2015)
15	pnnl2_lq	Leung and Qian (2009)
16	reid250	Reid et al. (2020)
17	reid500	Reid et al. (2020)
18	rutz	Rutz et al. (2014)
19	SAIL_v1	Experimental
20	shields	Shields and Kieh (2016a); Shields and Kiehl (2016b)
21	tempest_t2cntrl	McClenny et al. (2020); Ullrich and Zarzycki (2017); Rhoades et al. (2020)
22	walton	Experimental

Table S3. ARTMIP catalogs used here. Dataset is the name given on the ARTMIP website (https://www.earthsystemgrid.org/dataset/ucar.cgd.ccsm4.artmip.tier1.html). Where "experimental" appears in the reference column, it means that no corresponding peer-reviewed publication is yet available.

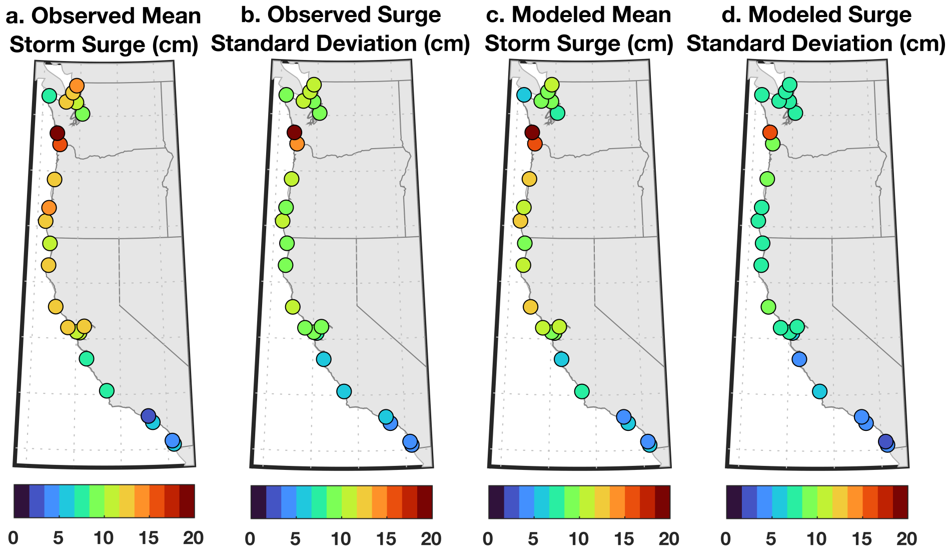

Figure S1. As in Figure 4 in the main text but based on the ECMWF Reanalysis Interim (Dee et al., 2011).

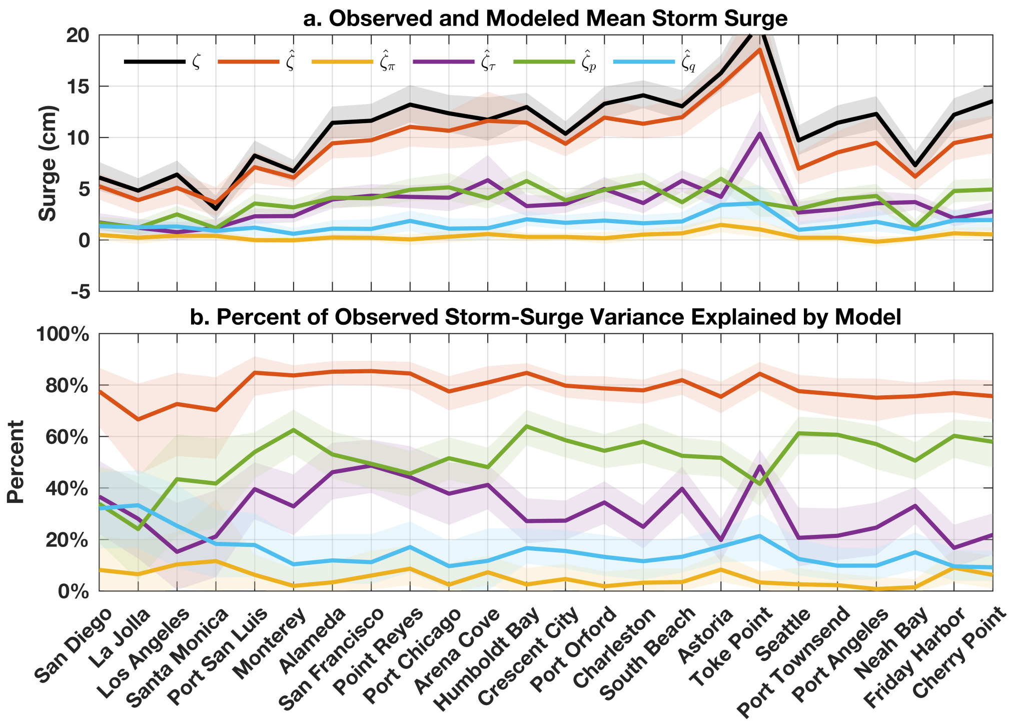

231 **Figure S2.** As in Figure 5 in the main text but based on the ECMWF Reanalysis Interim
232 (Dee et al., 2011).

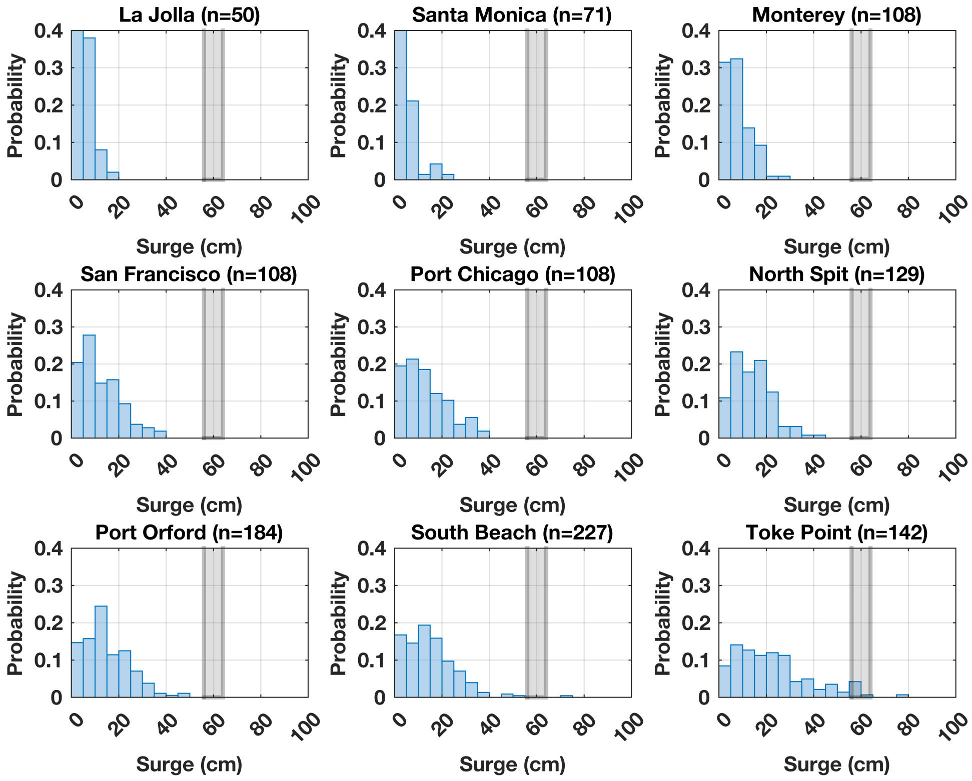

Figure S3. Blue shading shows probability density functions of surges during ARs at example tide gauges (location names and number of AR events identified in the title of each panel). For reference, gray shading identifies the 56–64-cm range that encompasses the HTF thresholds (above mean higher high water) at the tide gauges (cf. Table S1).

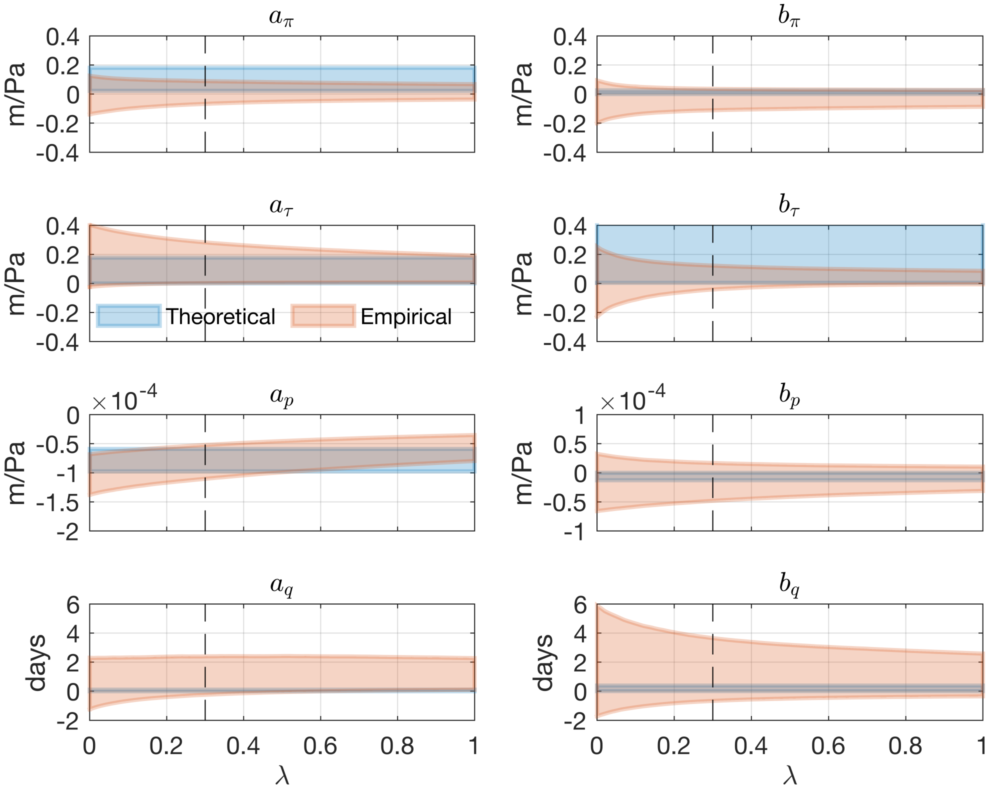

Figure S4. Coefficients between atmospheric forcing and storm surge ζ found empirically from regression analysis (orange) and expected theoretically from the analytical model (blue). Left column shows coefficients between ζ and atmospheric forcing [a's in Eqs. (1), (S16)–(S20)], whereas right column shows coefficients between ζ and the Hilbert transforms of atmospheric forcing [b's in Eqs. (1), (S16)–(S20)]. First row shows results for zonal wind stress π, second row meridional wind stress τ, third row barometric pressure p, and fourth row precipitation q. Empirical values are 95% confidence intervals across all sites as a function of ridge-regression parameter λ (vertical black dashes identify $\lambda = 0.3$). Theoretical values are shown as min/max ranges based on Eqs. (S16)–(S20) evaluated using parameter values/ranges in Table S2 and an angular frequency σ range between $2\pi/(1\text{ day})$ and $2\pi/(6\text{ days})$.

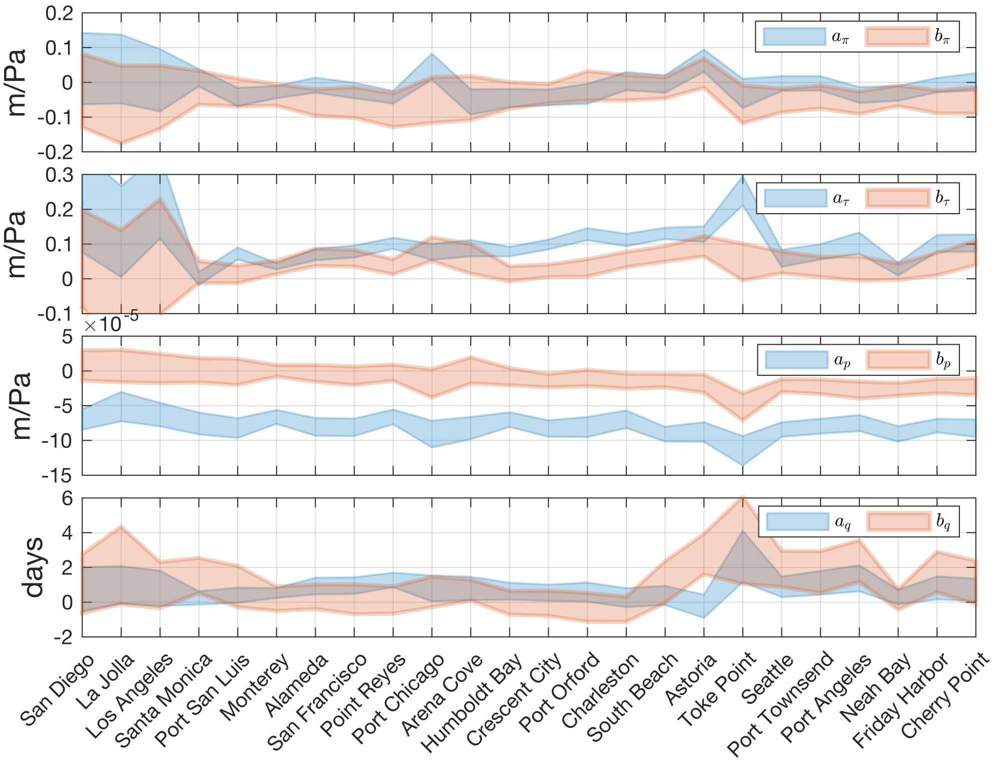

Figure S5. Coefficients between atmospheric forcing and storm surge ζ from regression analysis at each tide gauge. Blue shading identifies coefficients between ζ and atmospheric forcing [a's in Eqs. (1)] while orange shading identifies coefficients between ζ and the Hilbert transforms of atmospheric forcing [b's in Eqs. (1)]. First row shows results for zonal wind stress π, second row meridional wind stress τ, third row barometric pressure p, and fourth row precipitation q. Values are 95% confidence intervals and based on a ridge parameter $\lambda = 0.3$.

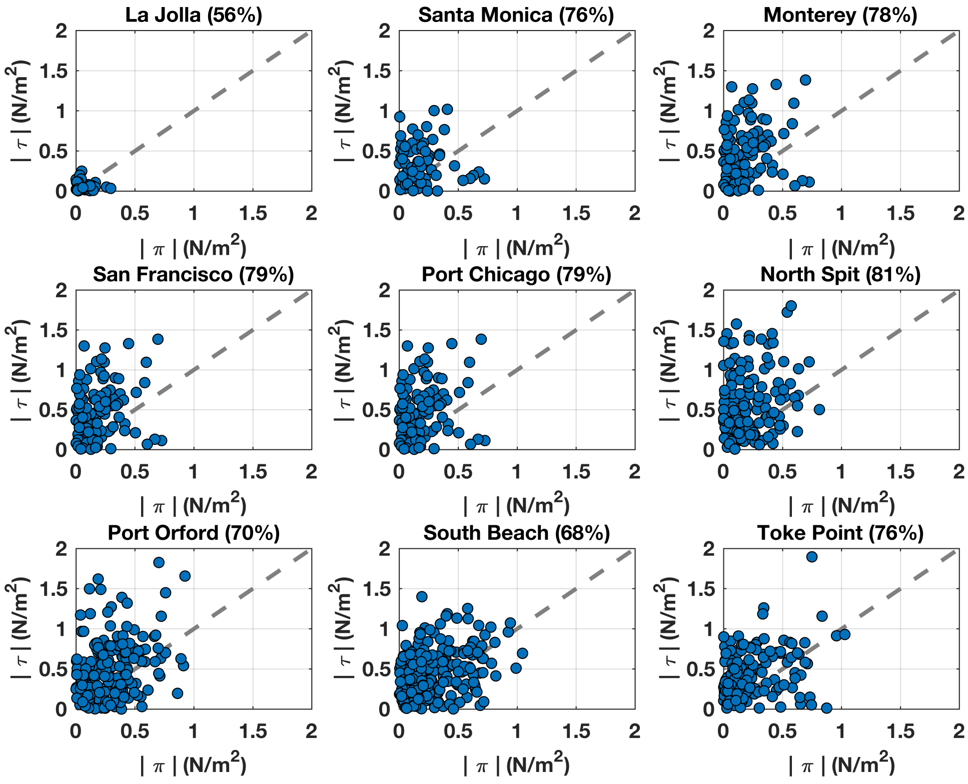

Figure S6. Blue dots compare magnitudes of high-pass-filtered anomalous daily zonal wind stress π (horizontal axes) and meridional wind stress τ (vertical axes) during ARs at example tide gauges (location names and percentages of ARs for which τ magnitudes are larger than π magnitudes identified in the title of each panel). For reference, grey dashes mark the 1:1 line.

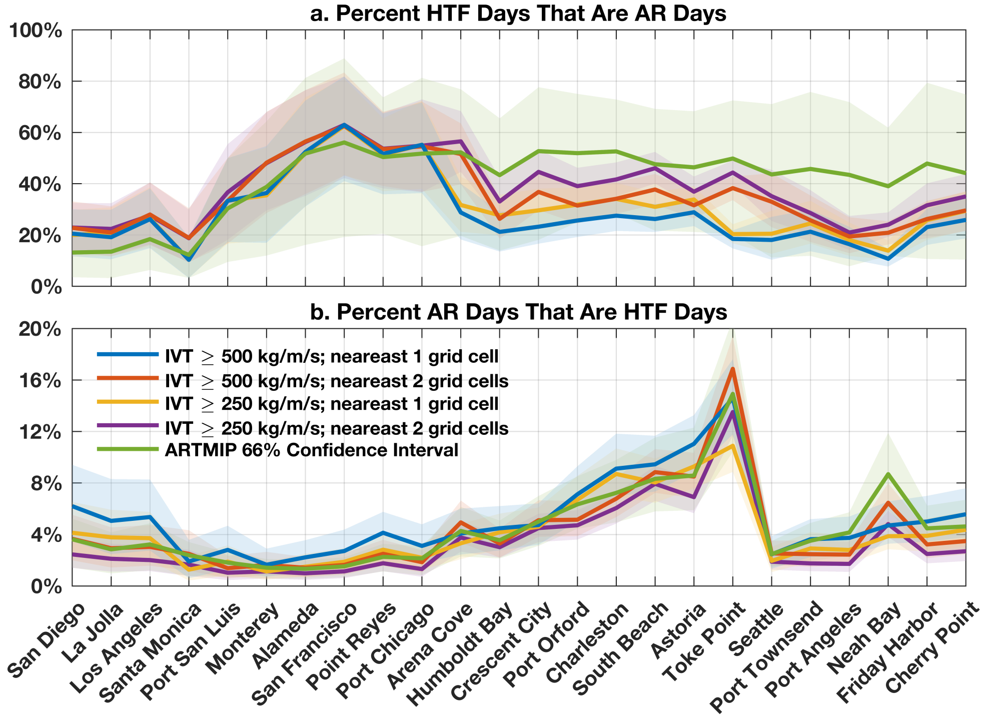

Figure S7. **(a)** Blue, orange, yellow, and purple curves and shading identify medians and 95% confidence intervals, respectively, for percent HTF days that are AR days at each tide gauge from the Gershunov et al. (2017) AR dataset and reproduced from Figure 3a in the main text. Green curve and shading are, respectively, best estimates and 66% confidence intervals based on the 22 other ARTMIP catalogs. **(b)** As in (a) but for percent AR days that are HTF days.
